# Manganese and Graphene Included Titanium Dioxide Composite Nanowires: Fabrication, Characterization and Enhanced Photocatalytic Activities

**DOI:** 10.3390/nano10030456

**Published:** 2020-03-04

**Authors:** Jun-Cheol Lee, Anantha-Iyengar Gopalan, Gopalan Saianand, Kwang-Pill Lee, Wha-Jung Kim

**Affiliations:** 1 Daegyeong Regional Infrastructure Technology Development Center, Kyungpook National University, Daegu 41566, Korea; uggenius@hanmail.net (J.-C.L.); algopal99@gmail.com (A.-I.G.); kplee@knu.ac.kr (K.-P.L.); 2 Faculty of Science, The University of Newcastle, Callaghan, NSW 2308, Australia; SaiAnand.Gopalan@newcastle.edu.au

**Keywords:** titanium dioxide nanowires, graphene and manganese inclusion, phase transformation, photocatalytic performance, synergistic effects

## Abstract

We report the detailed microstructural, morphological, optical and photocatalytic studies of graphene (G) and manganese (Mn) co-doped titanium dioxide nanowires (TiO_2_(G–Mn) NWs) prepared through facile combined electrospinning–hydrothermal processes. The as-prepared samples were thoroughly characterized using X-ray diffraction (XRD), transmission electron microscopy, X-ray photoelectron spectroscopy (XPS), Raman spectroscopy, and diffuse reflectance spectroscopy (DRS). XRD studies reveal the formation of mixed anatase-rutile phases or rutile phase depending on the dopant (Mn) precursor concentrations in the electrospinning dope and calcination temperature. The evaluation of lattice parameters revealed that the incorporation of Mn species and carbon atoms in to the lattice of anatase or rutile TiO_2_ could occur through substituting the sites of oxygen atoms. XPS results confirm the existence of Mn^2+^/Mn^3+^ within the TiO_2_ NW. Raman spectroscopy provides the evidence for structural modification because of the graphene inclusion in TiO_2_ NW. The optical band gap of G–Mn including TiO_2_ is much lower than pristine TiO_2_ as confirmed through UV-vis DRS. The photocatalytic activities were evaluated by nitric oxide (NOx) degradation tests under visible light irradiation. Superior catalytic activity was witnessed for rutile G–Mn-co-doped TiO_2_ NW over their anatase counterparts. The enhanced photocatalytic property was discussed based on the synergistic effects of doped G and Mn atoms and explained by plausible mechanisms.

## 1. Introduction

The breakthrough research report published by Fujishima and Honda on photocatalytic splitting of water on a titanium dioxide (TiO_2_) electrode under ultraviolet (UV) light irradiation forms the basis for extensive research inventions based on the photocatalysis phenomenon of TiO_2_. One of the limitations of TiO_2_ in photocatalytic applications is its large band gap (3.0–3.2 eV) because of the fact that TiO_2_ is photoactive only under UV irradiation [1]. The primary technical issues in terms of photocatalytic efficiency are the limited surface area of TiO_2_ and photoreaction [2]. Most of the electron–hole pairs undergo recombination within a few nanoseconds, and hence only a fraction of the charge carriers are involved in the photocatalytic reactions (∼10%) [3]. Considering these issues and the demands for future advanced applications of TiO_2_, there exists a need for the development of newly modified materials based on TiO_2_ having enhanced performances with respect to the photocatalytic properties. Strategies are being continuously evolved to modulate the properties of TiO_2_ and other metal oxides [4,5,6,7,8,9,10,11] towards achieving improved performances that include nanostructuring, generation of oxygen defects, chemical (metal or non-metal) doping, surface sensitization/modification, interface modification [12,13,14,15,16] and combining other semiconductors or other components to form nanocomposites/nanohybrids toward myriad of applications (energy, environment and health) [17,18,19,20].

The fabrication of TiO_2_ nanostructures with interesting morphologies and properties has attracted considerable attention in order to exploit their inherent physicochemical properties of TiO_2_ [21]. Many TiO_2_ nanostructured materials such as nanoparticles, exfoliated nanosheets, nanofibers, and nanowires (NW) have been developed through various synthesis methods [22]. Moreover, one dimensional (1D) semiconductor nanostructures can facilitate the light absorption and scattering, which is beneficial for photocatalytic reactions. Typically, 1D nanostructures of TiO_2_ (like NW) have been extensively studied because of their distinctive advantages such as high aspect ratio along with the typical features of TiO_2_ nanoparticles. In TiO_2_ NWs, the photogenerated carriers are expected to transfer along the axial direction effectively. In addition, the large specific surface area and chemical stability of 1D TiO_2_ allow them to be used as an ideal building block for assembling various surface heterostructures [23]. The prominent synthetic methods of TiO_2_ NW include sol–gel, hydrothermal and electrospinning. Among the various methods for the preparation of TiO_2_ NW, electrospinning is versatile and an up-scalable technique [24,25,26,27,28].

Researchers have been exploring methodologies to modify TiO_2_ for extending their optical response into the visible range [29]. Band engineering by incorporating impurities (either cations or anions) into TiO_2_ crystal structure, deposition of metal nanoparticles over the surface TiO_2_ particles, doping with anions/transition metals, sensitization by quantum dots, and formation of hybrids/ composites, etc., have been effectively utilized to extend the absorption of TiO_2_ from UV to the visible region. The impurity elements substitute the oxygen atoms from the lattice of TiO_2_ and decrease its band gap by generating sub energy levels between conduction and valence bands [30,31]. Doping TiO_2_ also changes their optoelectronic properties, facilitates separation of photogenerated charge carriers and leads to enhanced reaction rates for photocatalytic processes [32,33]. Non-metal (carbon, nitrogen, sulphur) doping has been extensively studied, especially after the work by Asahi et al. [34] on nitrogen-doped TiO_2_ [34,35]. The composites or hybrids of TiO_2_ with either graphene oxide [36], reduced graphene oxide, exfoliated graphene (G) sheets or G sheets having oxygenous functional groups (such as carboxylic (–COOH), hydroxyl (–OH) and carbonyl (C=O) groups) on the surface were effectively used to enhance the photocatalytic ability of TiO_2_ [24,37,38,39,40,41]. Through various studies, its understood that G inclusion into TiO_2_ improves the photocatalytic efficiency of TiO_2_ owing to its large specific surface area that can facilitate the distribution of TiO_2_ and enable narrowing of the band gaps of TiO_2_ [24,25,26,27,28,41,42]. Besides, the enhancement of photoactivity of TiO_2_ is ascribed to the synergistic effects between graphene oxide and/or G nanosheets and TiO_2_ [43,44].

Several reports revealed that doping of TiO_2_ with metals and/or non-metals minimizes the electron–hole recombination and increases its visible-light harvesting potential and these aspects have proved to be effective for accentuating the photocatalytic efficiency of TiO_2_ [45,46]. Among the metal doping for TiO_2_, manganese (Mn) doping for the modification of TiO_2_ attracts paramount interest because of the abundant nature of Mn, the possibility to extend the optical absorption in the visible or even the infrared solar light via the combined effects of narrowing the band gap, and the generation of internal band levels within the forbidden gap [47]. Oxygen vacancy and associated substitutional Mn in TiO_2_ crystal structure generate localized defect levels in the forbidden band, induce significant spin polarization and effectively reduce the energy [48]. To the best of our knowledge, reports on Mn doping into graphene including titanate composite NWs have not been attempted.

Extensive researches on single element doping of TiO_2_ in the past decades revealed that doping could improve the photoactivity of the catalysts only to some extent and has problems such as thermal stability and charge carrier trapping. In the process of further improving the photocatalytic activity more than single element doping, co-doped TiO_2_ were prepared through a judicial metal combination [49,50], metal non-metal elements [51,52] and double metal ions [53,54]. Kudo et al. reported that rutile TiO_2_ co-doped with chromium and antimony (TiO_2_:Cr/Sb), rhodium and antimony (TiO_2_:Rh/Sb), and nickel and tantalum or niobium (TiO_2_:Ni/(Ta, Nb)) showed visible light response to photocatalytic oxygen evolution [55,56]. La–Na co-doped TiO_2_ nanoparticles have been synthesized by a solvent-controlled, sol-gel route [57]. Several composite catalysts based on C, N and P co-doped TiO_2_ were prepared by sol-gel method [58]. Simultaneous doping of TiO_2_ using anions (C, N, etc.) and transition metals (Cr, V, Mo, etc.) results in significant narrowing of the bandgap. In such a situation, improvements in solubility limit of the dopant pair result from the charge states of p and n-type sites in non-compensated dopants (for example: Cr/N) [59]. Adopting this strategy, Cr–N co-doped TiO_2_ (TiO_2_:Cr, N) nanoparticles and single-crystal anatase [60] or rutile [61] thin films have been fabricated, having a diminished bandgap. It is understood that the interfacial charge-transfer processes are influenced by the presence of a metal co-catalyst or surface-bound molecular relays. Literature reveals that there are no reports on the preparation of Mn and graphene co-doped TiO_2_ NW.

In this work, we demonstrate a facile electrospinning and hydrothermal two-step methodology for the synthesis of graphene (G) and Mn species co-included with TiO_2_ nanowires (T(G–Mn) NW) as a potential photocatalytic material for NOx removal. These aspects have not been covered till date. It is worth noting that the hydrothermal stage guarantees the simultaneous inclusion of G and Mn species into the TiO_2_ NWs. Though the preparation of vertically aligned rutile TiO_2_ NW arrays has been reported [62], the reports on the preparation of rutile TiO_2_ NW co-doped with carbon and metal are scarce. Herein, we report a convenient method to prepare both anatase-rutile mixed phase T(G–Mn) NW and the pure rutile T(G–Mn) NW by controlling the annealing temperature. The morphology, structure and optical properties of T(G–Mn) NW prepared under different conditions were thoroughly characterized by various advanced techniques. The photocatalytic activities in NOx oxidation under visible light irradiation were comparatively investigated between the G and Mn including anatase and rutile NW. In addition, the photocatalysis mechanisms were explored.

## 2. Materials and Methods

### 2.1. Materials

Acetone (94.0%) and ethanol (99.5%) were obtained from OCI, Korea. Titanium isopropoxide (TIIP) (97%), manganese acetate tetrahydrate (99%), polyvinylpyrrolidone (molecular weight: 40,000 and 1.30 × 10^6^), and graphene oxide (15–20 sheets, 40–10% edge oxidized) were obtained from Sigma Aldrich, Seoul, Korea.

### 2.2. Preparation of G Included TiO_2_ Composite NWs

The two major steps involved in the synthesis include (i) preparation of G included TiO_2_ composites (titanate NWs) and (ii) calcination. The detailed preparation procedures of G-incorporated composite titanate nanofibers can be referred from our earlier reports published elsewhere [24,25,26,27]. A similar route has been adopted for the synthesis of Mn-embedded composite titanate NWs.

### 2.3. Preparation of Mn Included TiO_2_ Composite NWs

A typical experimental procedure is outlined here. Graphene (80 mg) and manganese acetate (40 mg) were dispersed in a 20 mL solution comprised of acetic acid and ethanol in the ratio of 3:7. The mixture was stirred well for 20 min. About 1.5 g of TIP was added slowly and stirred further for 30 min. Then, PVP with 40,000 and 1.30 × 10^6^ molecular weights were individually added in the ratio of 6:1 and stirred for 1 h. The entire mixture was allowed to stand for 6 h and left undisturbed. The prepared electrospinning dope solution was loaded into plastic syringes (10 mL) and electrospinning was performed with a high-voltage power of 25 kV. The electrospun G–Mn included titanate (TP(G–Mn) composite nanofiber mats were collected. The fiber mats were suspended in 20 mL of ethanol/water solution (50/50), and the hydrothermal reaction was carried out at 180 C for 2 h prior to calcination. The TP(G–Mn) composite nanofiber mats were thus prepared with different Mn loaded and designated (Table 1).

TP(G–Mn) composite nanofiber mats were calcined at 550 °C for 2 h in order to decompose PVP to obtain G–Mn including TiO_2_ NWs. Similarly, TP(G–Mn) composite nanofiber mats were calcined at 800 °C for 2 h to obtain G–Mn rutile TiO_2_ NWs. The calcinations were primarily performed to tune phase compositions of TiO_2_. The samples are designated based on the Mn precursor used in the electrospinning dope and the calcination temperature (Table 1).

### 2.4. Characterization

The morphology of the samples was examined by field-emission scanning electron microscopy (FE-SEM, SNE-3200M, SEC, Suwon, Korea) and field-emission transmission electron microscopy (FE-TEM, Titan G2 ChemiSTEM Cs Probe, FEI Company, Hillsboro, OR, USA). The crystal phases of the samples were examined by X-ray diffraction analyzer (D/Max-2500, Tokyo, Rigaku, Japan). The scanning angle 2θ was varied from 5° to 70° with a step size of 0.02 and a dwell time of 1.5 sec. The working voltage applied electric current and Cu Kα radiation were 40 kV, 200 mA and 1.5406 Å, respectively. The band gaps of the samples were determined by UV-Vis diffuse reflectance spectroscopy (S-3100, Seoul, Scinco, Korea) in the wavelength region 200 to 700 nm. X-ray photoelectron spectrum (XPS) of samples were recoded with an X-ray Photoelectron Spectroscopy (NEXSA, ThermoFisher, Waltham, MA, USA) using a Al-Kα monochromator source (1486.6 eV). Raman spectra of samples were obtained in the range 100 to 2,700 cm-1 using a Raman spectrometer (inVia reflex, Reinshaw, Wotton-under-Edge, UK), equipped with a 536 nm laser.

### 2.5. Photocatalytic Experiments

The photodegradation of NOx experiments was carried out under visible light irradiation by the gas-bag A method standardized by the Korea Photocatalyst Association. About 0.5 g of the photocatalyst was dispersed in distilled water to obtain a slurry. The photocatalyst slurry was applied on a 10 cm × 10 cm glass plate and dried for 1 h at 100 °C. Then, the photocatalyst-coated glass plate was then placed in a Tedlar bag (3 L volume) and filled with diluted nitrogen oxide gas using air. The nitrogen oxide concentration was kept as 1 vol. The Tedlar bag loaded with the sample and the gas was placed in a stainless-steel box. The sample was irradiated with the fluorescent lamp (Kumho Electric INC, three wavelength day light color, 17 W) fixed within the Tedlar box. The sample surface was irradiated with an illuminance of 3450 Lumen/m^2^. The concentration of NOx was measured with a NOx analyzer (GV-100s, Gastec) every hour for 6 h.

## 3. Results and Discussion

### 3.1. Phase Composition and Crystal Structure

**Figure 1:**
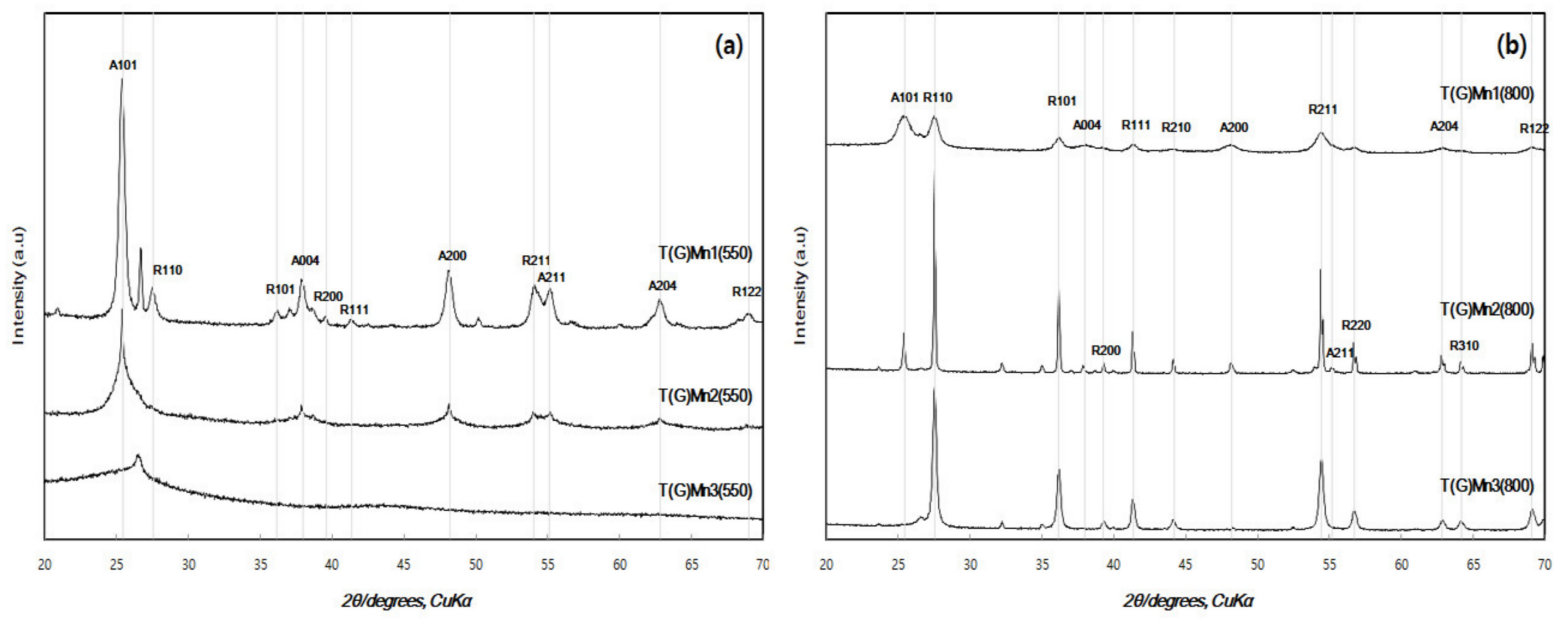
X-ray diffraction patterns of (a) T(G–Mn1)(550) NW, T(G–Mn2)(550) NW, and T(G–Mn3)(550) NW; and (b) T(G–Mn1)(800) NW, T(G–Mn2)(800) NW, and T(G–Mn1)(800) NW.

Figure 1 presents the XRD results of various TiO_2_(G–Mn) samples after annealing at different temperatures (550 °C and 800 °C) (Table 1). The TiO_2_(G–Mn) samples annealed at 550 °C exhibit the formation of the mixed anatase and rutile phases. The XRD patterns for the T(G–Mn1) and T(G–Mn2) samples sintered at 550 °C show anatase crystalline diffraction peaks located at 2θ = 25.3, 37.8, 48.1, 55.1, and 62.7 that correspond to the polycrystalline tetragonal (101), (004), (200), (211), and (204), reflections, respectively. In addition, the rutile phase reflections were identified by the diffraction peaks located at 2θ = 27.5, 36.1, 39.2, 41.4, 54.1, and 68.9 and indexed as (110), (101), (200), (111), (211), and (122), respectively. On the other hand, the XRD pattern of T(G–Mn3) sample sintered at 550 °C did not show any crystalline pattern but rather presents an amorphous pattern with a broad peak around 2θ = 26.4. The formation of amorphous phase anatase is a rather interesting feature to note. The amorphous TiO_2_ is expected to be electronically defective in nature and can be attributed to the reduction of Ti^4+^ cations to the Ti^3+^ state, through the probable n-type doping, where tetravalent Ti may be substituted by polyvalent M cation [63]. When Mn concentrations are higher (120 mg in the electrospinning dope, Table 1), this possibility of amorphous anatase formation arises. Alternatively, the modification of the bond structure by creating O vacancies or Ti^3+^ interstitials is expected [63]. Hence, we believe that the reduction of the Ti coordination number and the shortening of the Ti–O bond could be the reason for amorphous TiO_2_ transformation in the case of the T(G–Mn3) (550) sample. The XRD pattern of 800 °C annealed samples predominately exhibit the existence of tetragonal rutile phase reflections. The sample T(G–MN1)(800) contains a very weak (101) anatase reflection peak. The position of the diffraction peaks is in good agreement with those given in ASTM data card (#21-1272) for anatase and ASTM data card (#21-1276) for rutile. It is interesting to inform that there are no reflection peaks that correspond to manganese phases in the XRD patterns, even with the highest doping concentration (Table 1). It must be noted that characterization results of the following samples prepared under similar experimental conditions were already reported in our earlier work [24] and hence are not presented here.

The percentage (%) of anatase and rutile phases in the samples was calculated and presented in Table 1. For the sake of comparison, the percentage anatase and rutile phases in the TiO_2_ samples without G and Mn (T(550) and T(800)) and samples prepared without including Mn precursor in the electrospinning stage (T(G)(550) and T(G)(800) are also presented in Table 2. The T(550) and T(G)(550) comprise mixtures of anatase and rutile phases, with rich anatase phase predominance (~90%). The T(G) (550) sample contains slightly higher anatase (93.76%) as compared to T(550) (90.14%). However, the 800 °C annealed samples (T(800)) and T(G) (800) correspond to nearly pure rutile phases (Table 1). Scherrer’s formula (see experimental part) was used to calculate the average crystalline size (D) by using the diffraction peak (101) for anatase phase and the peak (110) for rutile phase with λ = 1.54056 × 10^−10^ m. These D values are shown in Table 2. The D spacings were found to have variations based on the annealing temperature and amount of Mn dopant (Table 2). No correlations could be deduced between the Mn dopant concentration and D values. The amorphous–anatase–rutile phase transition of TiO_2_ nanoparticles was found to depend on their sizes [64]. As a common guideline, the anatase-rutile phase transition temperature is substantially lower for smaller TiO_2_ particles, and crystalline rutile phase formation is dominant at temperatures exceeding 550 °C [65]. In our experiments, the calcination of the samples at 800 °C resulted in nearly complete rutile phase formation. (Table 2)

Table 2 presents the comparison of lattice parameters (d-spacing, unit cell values and unit cell volume) for G–Mn co-doped, G-doped and undoped TiO_2_ samples. The (101) and (200) diffraction peaks, respectively at 25.3° and 48.1°, were used to calculate the a and c lattice parameters of tetragonal anatase, as well as the unit-cell volumes. Similarly, the diffraction peaks that correspond to (101) and (200) reflection planes at 36.1° and 39.2° were used to calculate the lattice parameters of tetragonal rutile and unit-cell volumes. The full width half maximum values of the (101) lattice reflections of the anatase and rutile phases are presented in Table 2. For the 550 °C calcined samples (T(G–Mn1(550)), T(G–Mn2(550)), T(G)(550), and T(550), the lattice parameters correspond to tetragonal crystal lattice with a = b ≠ c (Table 1), respectively. The sample T(G–Mn3(550) is amorphous. The 800 °C annealed samples (T(G–Mn1(800)), T(G–Mn2(800)), T(G–Mn3(800), T(G)(800), and T(800))) retain the tetragonal structure with a = b ≠ c, however, they have distinctly different a= b and c values (Table 1). As compared to T(550) and T(G)(550), the c values of the T(G–Mn1)(550) and T(G–Mn2)(550) increased from 9.499 A to 9.867 A. The ionic radii of the Mn species are 0.079 nm (Mn^3+^, high spin), 0.072 nm (Mn^3+^, low spin), and 0.067 nm (Mn^4+^) [66]. The ionic radius of Ti^4+^ in anatase structure was found to be 0.075 nm. The Mn ionic radii are within the threshold for possible extensive solid solution formation and TiO_2_ structure disruption [67]. The inclusion of Mn species can be the reason for the elongation of the c-axis of the tetragonal TiO_2_ anatase structure. Similarly, the c values of T(G–Mn1)(800) and T(G–Mn2)(800) are larger (4.564 A) as compared to the c values of T(800) and T(G)(800)(2.959 A). Hence, a distortion of rutile tetragonal structure is evident through the elongation of the c-axis. On comparing the ionic radii of Mn^3+^ and Mn^4+^, the substitution of Ti^4+^ by Mn^4+^ is easier while substitution by Mn^3+^ requires the formation of oxygen vacancies for charge balance, and in turn is advantageous for the photocatalytic activity [68]. We believe that increased oxygen vacancies could be brought about by the substitution of Ti by lower valence Mn ions [69]. The generation of oxygen vacancies lattice can reduce the constraint and facilitate the reconstructive phase transformation. When the annealing temperature was increased from 550 °C to 800 °C, the reconstruction of anatase to rutile transformation happened through the rearrangement of the TiO_2_ lattice. We compared the unit cell volume between the 500 °C and 800 °C annealed samples. Nearly 54% decrease in unit cell volume was witnessed between 550 °C and 800 °C annealed pure TiO_2_ samples (Table 1). The decrease in unit cell volume upon anatase to rutile transformation is in accordance with the literature [35,70]. There is not much difference in unit cell volume between T(800) and T(G)(800) samples, signifying that the inclusion of G did not alter much the TiO_2_ crystal structure. On the other hand, the inclusion of Mn along with G was accompanied with an increase of ~35% unit cell volume. The atomic status of Mn in the TiO_2_ structure is further explored through XPS analysis (latter part of discussion).

### 3.2. Probing Morphology Through SEM, TEM and HR-TEM

**Figure 2:**
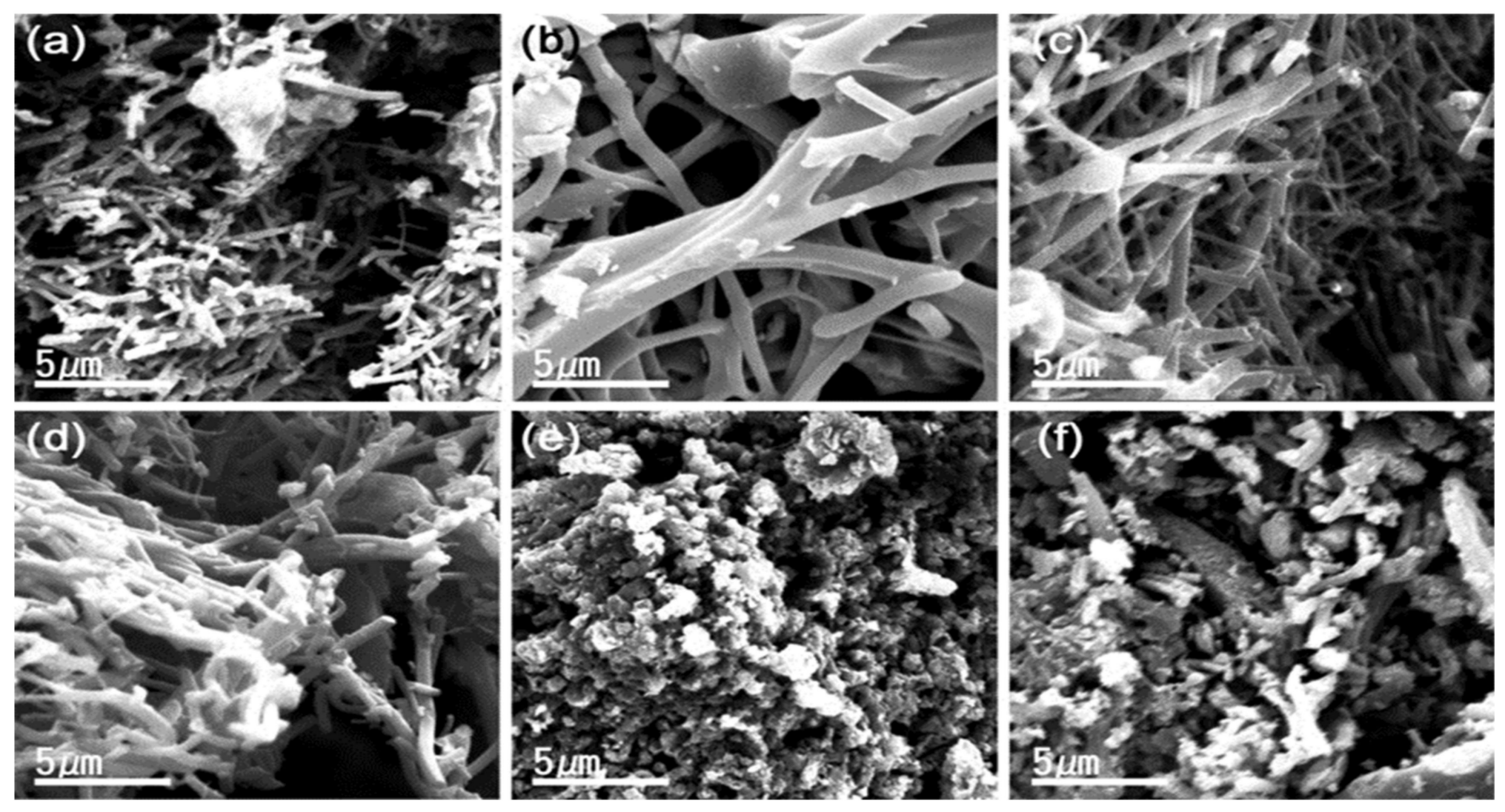
FE-SEM images of (**a**) T(G–Mn1)(550) NW, (**b**) T(G–Mn2)(550) NW, (**c**) T(G–Mn3)(550) NW, (**d**) T(G–Mn1)(800) NW, (**e**) (a) T(G–Mn2)(800) NW, and (**f**) T(G–Mn1)(800) NW.

While, there is an increasing trend in the nanofiber diameter and length with an increase in Mn amount in the electrospinning precursor dope, between TP(G–Mn1) and TP(G–Mn2), a drastic decrease in diameter and length was witnessed for TP(G–Mn3) (Supplementary Material, Figure S1a–c). We believe that the inclusion of Mn in the titanate structure could be the reason for the variations in the diameter and length of the nanofibers. Figure 2 depicts the FE-SEM images of T(G–Mn1)(550) NW, (b) T(G–Mn2)(550) NW, (c) T(G–Mn3)(550) NW, (d) T(G–Mn1)(800) NW, (e) T(G–Mn2)(800) NW, and (f) T(G–Mn1)(800) NW. The variations in the lattice parameters of the calcined samples (Table 1) partially justify our proposal. FE-SEM images of 550 °C and 800 °C (Figure 2) calcined samples exhibit NW morphology with a decrease in diameter and length of NWs. To note, 800 °C calcined P(G–Mn3) showed the lowest the diameter and length amongst the G–Mn including TiO_2_ NWs.

**Figure 3:**
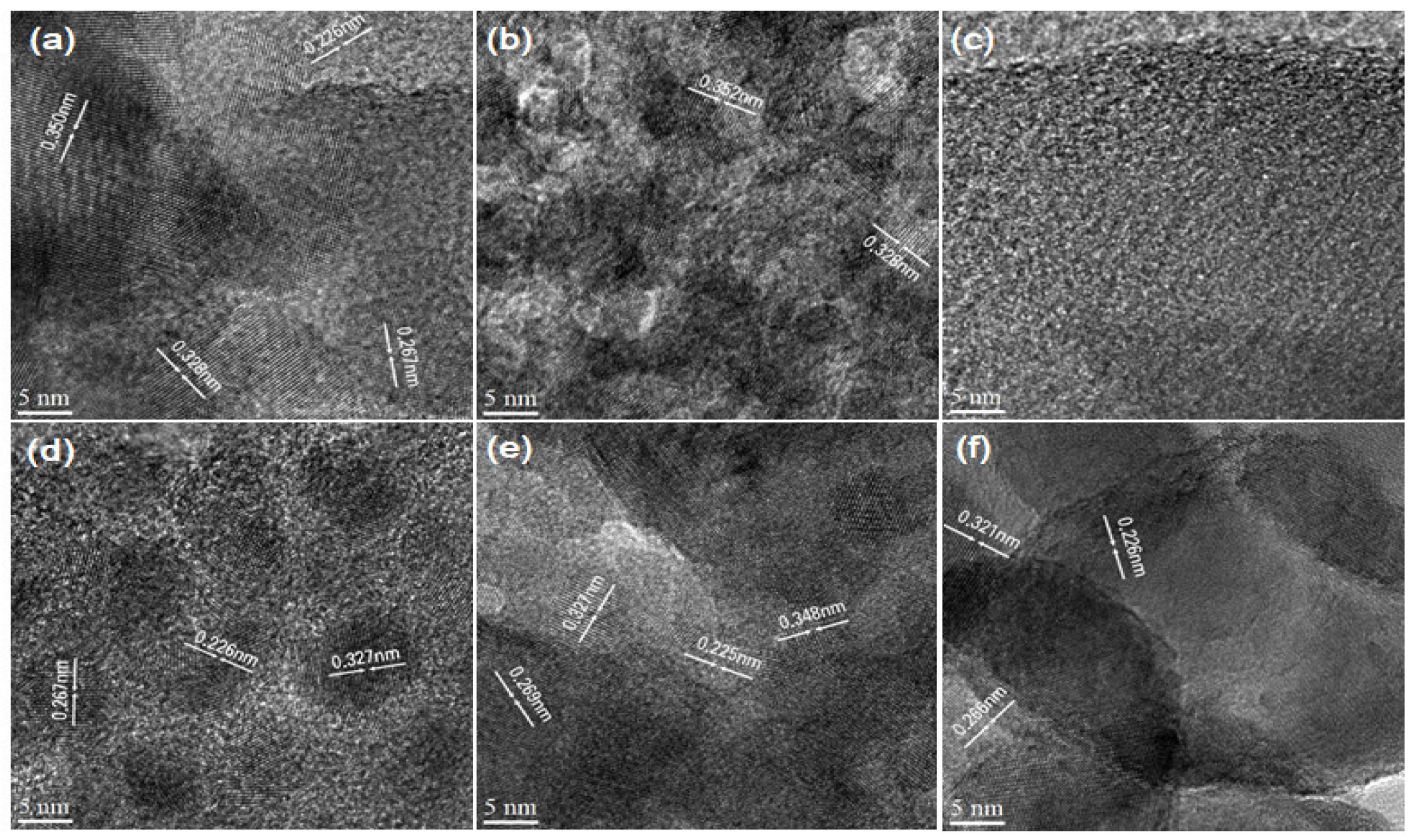
HR-TEM images of (**a**) T(G–Mn1)(550) NW, (**b**) T(G–Mn2)(550) NW, (**c**) T(G–Mn3)(550) NW, (**d**) T(G–Mn1)(800) NW, (**e**) (a) T(G–Mn2)(800) NW, and (**f**) T(G–Mn1)(800) NW showing the lattice fringes.

TEM (Supplementary Material, Figure S2a–f) and HR-TEM (Figure 3a–f) images were further acquired to probe the NWs morphologies and crystal structures. TEM images of single NWs of 550 °C annealed samples, T(G)Mn1(550), T(G)Mn2(550), and T(G)Mn3(550) (Supplementary Material, Figure S2a–f) inform us that surface of NW is smooth with variations in the diameters. While the diameter of T(G)Mn2(550) NW is larger (~790 nm) than T(G)Mn1 (500) NW (~270 nm), the diameter of T(G)Mn3(550) is much decreased to ~150 nm. HR-TEM images of T(G)Mn1(550), T(G)Mn2(550) and T(G)Mn3(550) inform that T(G)Mn1(550) and T(G)Mn2(550) are polycrystalline and T(G)Mn1(550) and T(G)Mn3(550) are amorphous (Figure 3). The amorphous characteristics of T(G)Mn3(550) are evident from the TEM (Supplementary Material, Figure S2a–f) and very well corroborate XRD (Figure 1). The distance between the adjacent lattice fringes are investigated for the polycrystalline samples to ascertain the lattice plane of the as-developed NWs. HR-TEM images of T(G)Mn1(550) and T(G)Mn2(550) showed lattice spacing of d = 0.354 nm for the (101) plane of the anatase phase, and d = 0.325 A is assigned to the interplanar distance of the rutile phase of the (110) plane. Thus, T(G)Mn1(550) and T(G)Mn2(550) comprised of anatase-rutile mixed phases (Figure 3) (Table 2).

**Figure 4:**
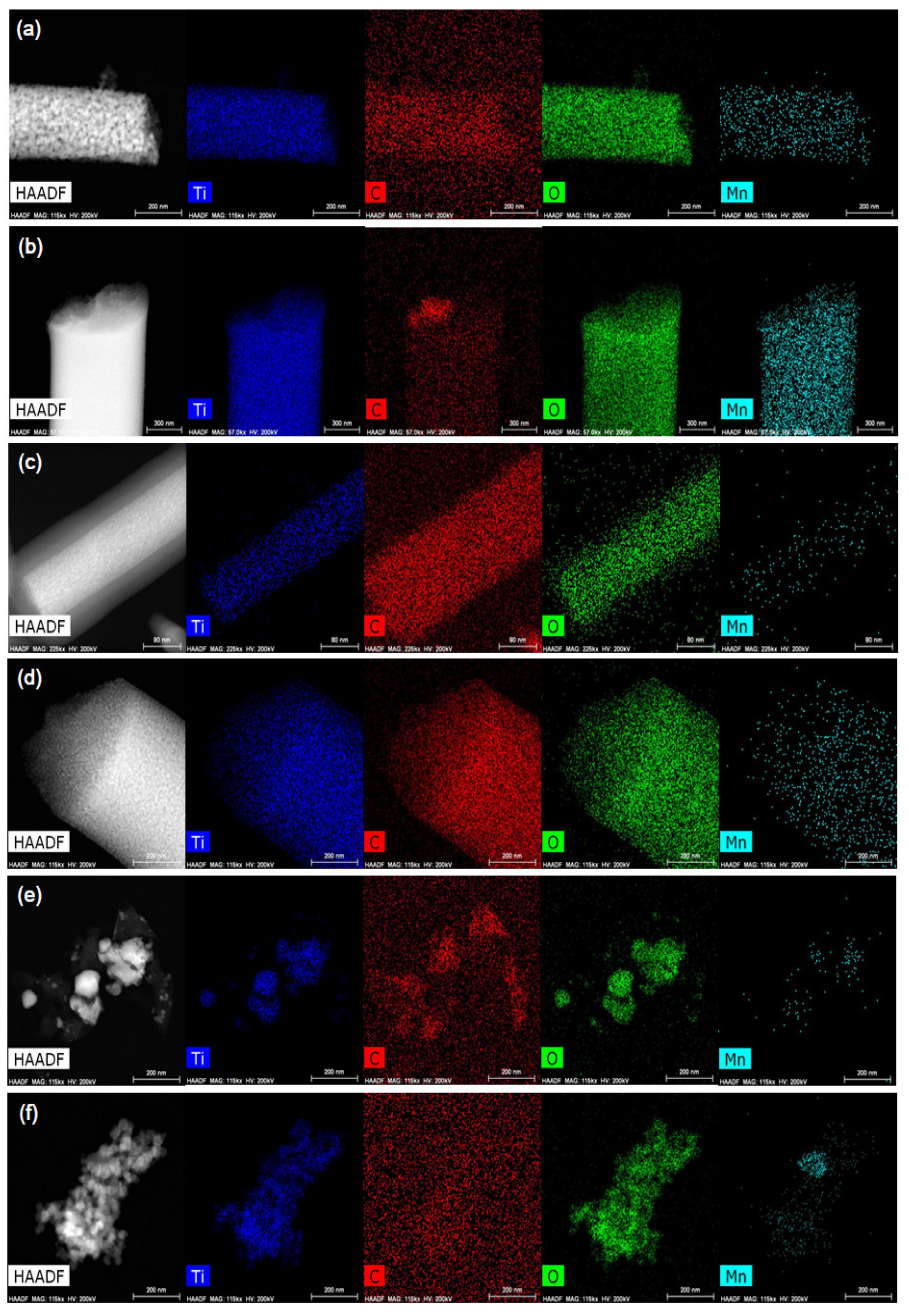
Elemental mapping for Ti, C, O, and Mn as deduced from HR-TEM images of (**a**) T(G–Mn1)(550) NW, (**b**) T(G–Mn2)(550) NW, (**c**) T(G–Mn3)(550) NW, (**d**) T(G–Mn1)(800) NW, (**e**) (a) T(G–Mn2)(800) NW, and (**f**) T(G–Mn1)(800) NW.

The elemental distribution within the NWs are presented in Figure 4. One can notice that both carbon (graphene) and Mn species are distributed within the NWs. However, there is a difference in Mn distribution throughout the NWs. While Mn species are densely distributed within the NWs, in the case of T(G)Mn1(550) and T(G)Mn2(550), Mn species are randomly distributed in T(G)Mn3 (550) NWs with a lesser density of distribution (Figure 4). A similar trend can be seen in the distribution of oxygen species within the NWs. We presume that the inclusion of Mn within the NW using different amounts of Mn precursors (Table 2) alters the structure of TiO_2_. Figure 4 presents the Ti, O, C, and Mn elemental mappings of the 800 °C annealed samples, (TG)Mn1(800), T(G)Mn2(800) and T(G)Mn3(800). TEM images of the single NW of the (TG)Mn1(800) shows the formation of longer NW(few µm long) with a diameter of ~560 nm and smooth surfaces (Supplementary Material, Figure S2a–f). On comparing the diameter of the T(G)Mn1(500) NW (~270 nm) annealed at 550 °C, the diameter of T(G)Mn1(800) increased to 560 nm. However, TEM images of (TG)Mn2(800) and (TG)Mn3(800) show the presence of shorter NWs. Hence, we believe that heat treatment at an elevated temperature causes collapse in the structure of the NWs. HR-TEM of T(G)Mn1(800), T(G)Mn2(800), and T(G)Mn3(800) showed lattice spacing of d = 0.325 nm for the (001) plane and 0.267 nm for the plane of the rutile phase (Figure 3). Thus, the 800 °C annealed TiO_2_ samples predominantly exhibited rutile phases, and the TEM observation confirms the anatase to rutile phase transformation as evident from XRD results (Table 1). The elemental distributions of Ti, O, C, and Mn in the NW of the samples are presented (Figure 4a–f). In conjunction with the results from TEM, HR-TEM and elemental mapping, we conclude that the length and diameter of the NWs; phase composition; and Ti, O, C, and Mn elemental distributions are dependent on calcination temperatures as well the amount of Mn precursor used in the fabrication procedure of the as-prepared photocatalysts (Table 2).

### 3.3. X-ray Photoelectron Spectroscopy (XPS), Raman and Diffuse Reflectance Spectra

Details pertaining to XPS, Raman and diffuse reflectance spectra are provided in the Supplementary Information. (XPS (Figures S3–S5), Raman (Figures S6 and S7) and diffuse reflectance (Figures S8 and S9).

### 3.4. Photocatalytic Performance

#### 3.4.1. NOx Removal

**Figure 5:**
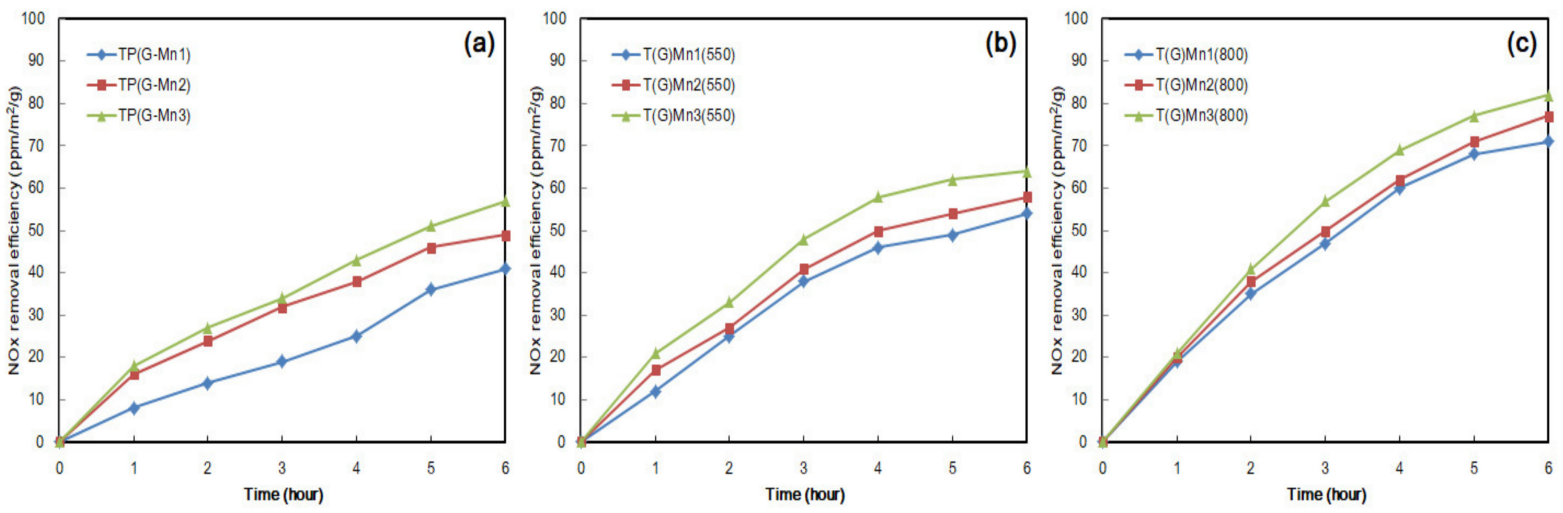
Photocatalytic NOx removal efficiency for (**a**) TP(G–Mn1), TP(G–Mn2) and TP(G–Mn3); (**b**) T(G–Mn1)(550) NW, T(G–Mn2)(550) NW, and T(G–Mn1)(550) NW; and (**c**) T(G–Mn1)(800) NW, T(G–Mn2)(800) NW, and T(G–Mn3)(800) NW. Photocatalytic experiments were performed three times and the results are represented in terms of error bar.

The photocatalytic degradation NOx of the as-prepared G–Mn co-doped samples was investigated under light irradiation and presented in Figure 5. The plots showing the NOx removal efficiency against irradiation time over different photocatalysts are presented in Figure 5. The photocatalytic NOx removal efficiency takes the following order: T(G)Mn3 (800) > T(G)Mn2 (800) > T(G)Mn1 (800) > T(G)Mn3 (550) > T(G)Mn2 (500) > T(G)Mn10) > TP(G–Mn3) > TP(G–Mn2) > TP(G–Mn1), respectively. The results informed that under the similar condition for G inclusion in the samples, both Mn concentrations used for the preparation and annealing temperature influence the photocatalytic NOx removal efficiency. Typically, increasing the Mn doping amount in the samples results in enhanced NOx removal efficiency. This trend could be seen among the non-annealed and their annealed counterparts. The NOx removal efficiency of TP(G–Mn3) was found to be 1.4 times higher than TP(G–Mn1). A similar trend could be witnessed between T(G)Mn3 (550) and T(G)Mn1(550), as well as between T(G)Mn3 (800) and T(G)Mn1 (800). A more quantitative comparison for the NOx removal efficiencies between the G and Mn co-doped samples was performed using the Langmuir–Hinshelwood model in the initial time period to evaluate the reaction rates of NOx photodegradation. Clearly, the 800 °C calcined samples show higher NOx removal efficiency as compared to 550 °C annealed samples. In conjunction with the confirmation from the XRD data that 800 °C annealed samples predominantly have a rutile structure (Table 1), we conclude that rutile phase along with G and Mn co-doping enhanced the photocatalytic efficiency. It must also be noted that G and Mn co-doped samples show better NOx removal performance as compared to pristine TiO_2_ and G-doped TiO_2_ samples.

**Figure 6:**
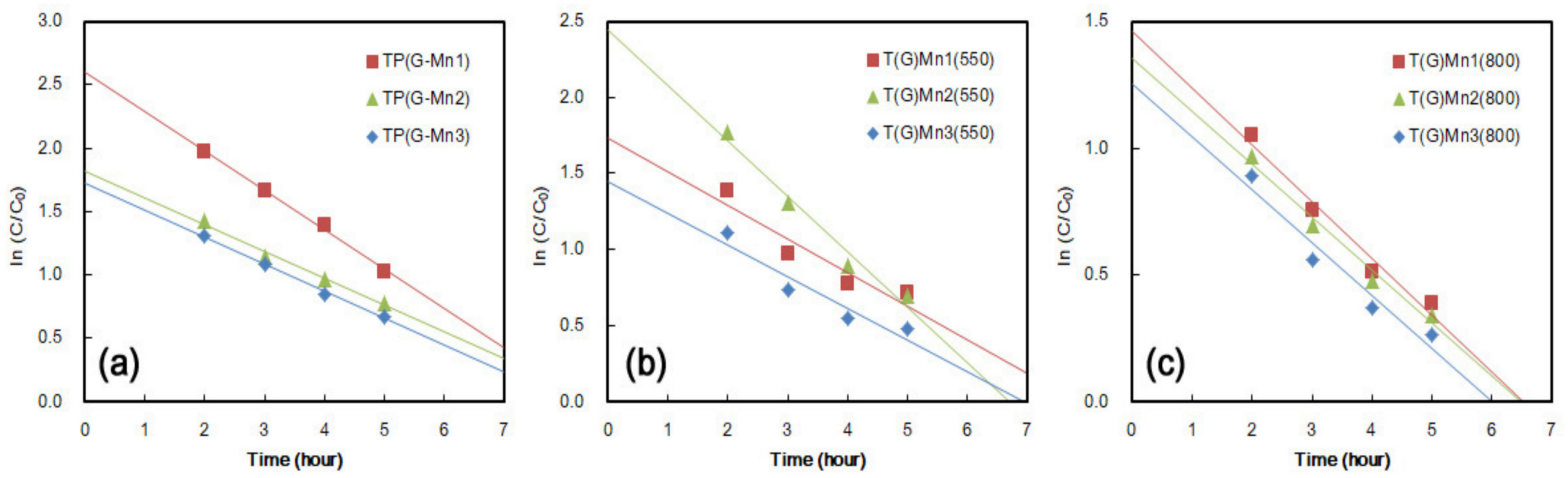
Kinetics of photocatalytic NOx degradation: (**a**) TP(G–Mn1), TP(G–Mn2) and TP(G–Mn3); (**b**) T(G–Mn2)(550) NW, T(G–Mn3)(550) NW, and T(G–Mn1)(550) NW; and (**c**) T(G–Mn1)(800) NW, T(G–Mn2)(800) NW and T(G–Mn1)(800) NW.

The photocatalytic degradation of NOx was found to follow mass transfer controlled first-order kinetics, as evidenced by the linear plot of ln(C/C_0_) versus photocatalytic reaction time t (Figure 6). We also compared the photocatalytic NOx degradation rate (mol/h) of the samples at 6 h, which followed the order: T(G)Mn3 (800) > T(G)Mn2 (800) > T(G)Mn1 (800) > T(G)Mn3 (550) > T(G)Mn2 (500) > T(G)Mn10) > TP(G–Mn3) > TP(G–Mn2) > TP(G–Mn1) with rates as 0.137, 0.128, 0.118, 0.107, 0.097, 0.095, 0.090, 0.082, and 0.068 μmol/h, respectively. It is commonly known that anatase is a better photocatalyst (compared to rutile and brookite) based on the photocatalytic application of TiO_2_ (with specific reference to the anatase crystalline phase) [71]. On the contrary, rutile TiO_2_ (r-TiO_2)_ has scarcely reported to be active for the photodegradation of organic compounds. However, a proportionate or significant photocatalytic activity has been exhibited by samples containing mixtures of anatase and rutile [72,73]. Nevertheless, r-TiO_2_ possess more advantageous characteristics than anatase such as high chemical inertness, superior light scattering characteristics and lower cost [74]. In this regard, Wang et al. reported the high photocatalytic activity of r-TiO_2_ for the decomposition of Rhodamine-B in water under artificial solar light irradiation [75]. Bacsa and Kiwi found that the presence of r-TiO_2_ showed enhanced catalytic activity compared to pure anatase TiO_2_ towards the degradation of p-coumaric acid [76]. The r-TiO_2_ has also been shown to be more active than anatase in photo-decomposition of H_2_S [77] and photo-oxidation of H_2_O with Fe^3+^ [78]. The reproducibility of the photocatalytic activity was also tested by measuring the photocatalytic activity of the similarly prepared photocatalyst (for three times). The experimental results are represented in terms of changes in the photocatalytic performances (Figure 5). While doing so, we could estimate the stability of the photocatalyst by performing three subsequent experiments by utilizing the same amount of sample. A decrease of ~5% photocatalytic activity was witnessed after three experiments. Among the investigated samples, T(G–Mn3) (800) NW showed superior photocatalytic performance.

On perusal of literature, the Mn–graphene co-doped TiO_2_ NW with rutile rich phase reported in this work exhibited superior photocatalytic NOx removal performance as compared to pristine TiO_2_ NWs and Mn-doped TiO_2_ materials, and thus, signifies the importance of Mn–G simultaneous co-doping and transformation to rutile TiO_2_.

#### 3.4.2. Plausible Mechanism

**Scheme 1:**
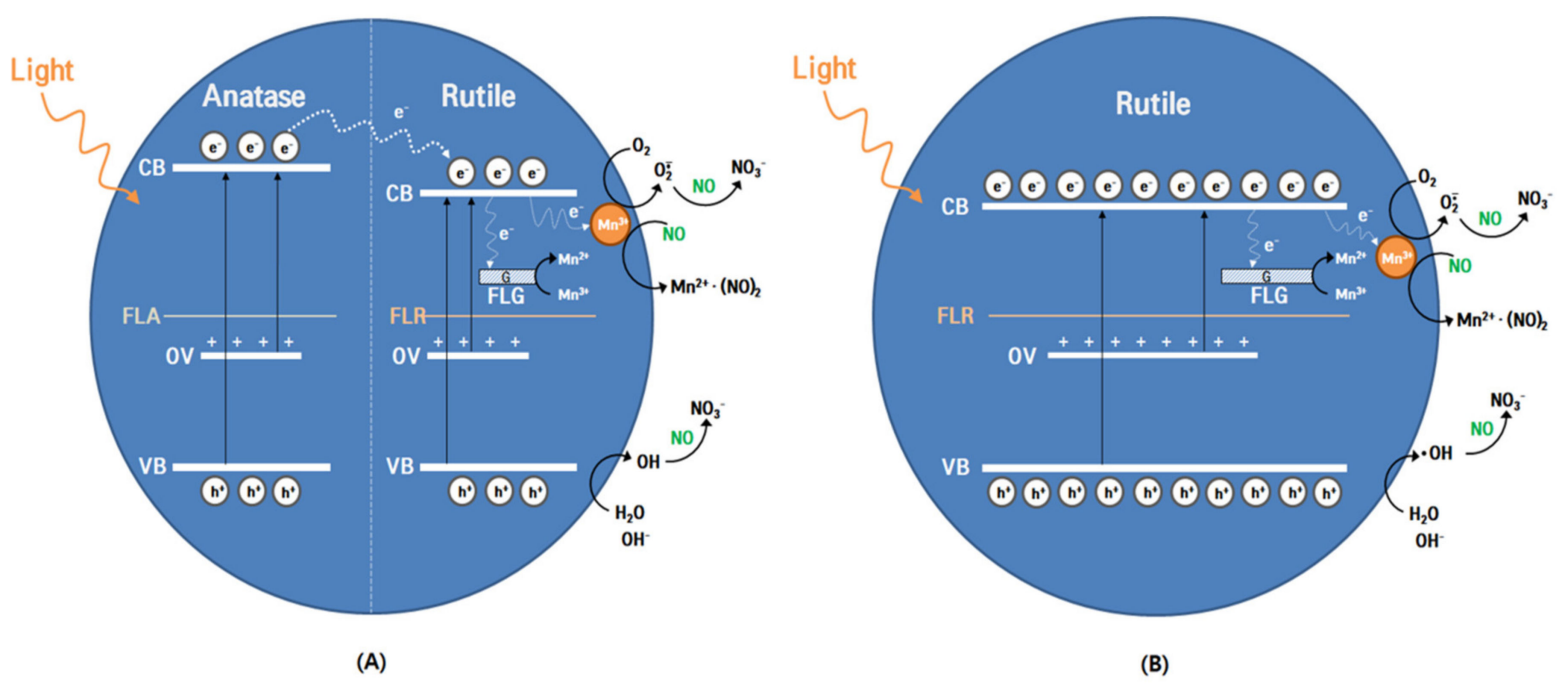
Plausible photocatalytic NOx degradation mechanisms for (**A**) anatase-rutile mixed phase T(G–Mn) NW and (**B**) rutile T(G–Mn) NW. (h: Holes, e: Electrons, CB: Conduction band, VB: Valence band, OV: Oxygen vacancy, FLA: Forbidden level anatase, FLR: Forbidden level rutile and FLG: Forbidden level band-gap).

In general, we believe the formation of TiO_2_ (anatase and rutile mixed phase upon annealing at 550 °C or pure rutile phase upon annealing at 800 °C) with oxygen vacancy as well as G–Mn co-doping has been found to boost the photocatalytic performance under visible-light irradiation. We propose a plausible mechanism for the photocatalytic NOx removal for the G–Mn co-doped and annealed at 550 °C and 800 °C, respectively (Scheme 1A,B). Firstly, we explain the mechanism for 550 °C annealed samples. When light irradiates on surface of the T(G)–Mn samples, both the anatase and the rutile TiO_2_ generate holes (h^+^) and electrons (e^−^) as presented in Scheme 1. Then the generated electrons are migrated from the valence band (VB) of TiO_2_ to conduction bands (CB) of the TiO_2_ anatase phase, and further the electrons are transferred to the CB of rutile TiO_2_. As the band gap of rutile TiO_2_ is smaller than anatase TiO_2_, the electrons from VB of rutile TiO_2_ could also be simultaneously excited to VB of rutile TiO_2_. The inter-state energy levels that are generated by oxygen vacancies (OV) can also populate the electron to the VB of anatase and rutile TiO_2_. The photogenerated electrons are expected to be efficiently trapped by Mn(III) sites to convert to Mn(II) states, and the Mn(III/II)-trapped electrons react towards atmospheric oxygen efficiently as the reaction can kinetically compensate the lower thermodynamic driving force for the reduction of oxygen; the NO species adsorbed at the Mn(II) sites (Mn^2+^(NO)_2_, ads) and re-oxidation of Mn^2+^ to Mn^3+^ through the formed hydroxyl radicals are expected to occur through the following reaction:

Mn^2+^(NO)2 + Ti − OH → Mn^3+^(NO)2, ads + Ti − OH (1)

The re-oxidation of Mn^2+^ to Mn^3+^ reduces the amount of π back bonding with NO and regenerating the catalytic activities. The formation of intermediates such as O_2_- and OH· can dramatically influence the photocatalytic performances of TiO_2_, which are dependent on the recombination rate of the bound (e–h) pairs. Therefore, the existence of an electron sink within a TiO_2_ lattice can promote the separation of the election-hole pairs and in turn can enhance the photocatalytic performance. G is proved to be a competent material because of its highest theoretical electron mobility. The photo-generated electrons in the CB of TiO_2_ can rapidly transport into G owing to a more positive Fermi level of the TiO_2_ [24,25,79]. Thus, electrons in the CB of TiO_2_ could interact with adsorbed O_2_, generating O_2−_, HO_2_ and OH radicals via one-step or multi-step reactions, and these active species enhance the NOx oxidation processes. Also, the residual holes in the VB of TiO_2_ or in the Mn^3+^ impurity level could interact with adsorbed water to produce OH radicals or directly react with NOx to form nitrate [80]. The holes are transferred from VB of anatase TiO_2_ to rutile TiO_2_, which in turn promote the charge separation efficiently and increase the lifetime of the charge carriers. Consequently, the photocatalytic activity is enhanced. The sequence of reactions that cause photocatalytic NOx degradation are presented below in terms of the G–Mn-mediated recombination mechanism for the photo-generated electrons and holes.

Light + TiO_2_− → e^−^ + h^+^ (2)

Ti(O2)ads + e−k1 − → Ti(O_2_^−^)ads (3)

Ti − OH^−^ + h+ → Ti − OH• (4)

Mn^3+^ + e−k3 − → Mn^2+^ (5)

Mn^2+^ + 2NO → Mn^2+^(NO)_2_,ads (6)

G + + e^−^ → G(e^−^) (7)

Mn^3+^(NO)_2_,ads→ Mn^3+^ + 2 NO(g) h^+^ + NO + 2H_2_O → NO_3_^–^ + 4H^+^ (8)

•O_2_^−^+NO → NO_3_ – (9)

h^+^ + H_2_O → •OH (10)

2•OH + NO → NO_2_ + H_2_O (11)

NO_2_ + •OH → NO_3_^–^ (12)

Mn + (NO)_2_ + Ti − OH• → Mn^3+^(NO)_2_,ads + Ti − OH^−^ (13)

The difference between the reaction mechanisms for 550 °C annealed and 800 °C annealed G–Mn-co-doped TiO_2_ is illustrated in Scheme 1B. What is intriguing about the difference is the fact that 800 °C annealed G–Mn-co-doped TiO_2_ exhibited higher photocatalytic performance as compared to 550 °C annealed G–Mn-co-doped TiO_2_. While TiO_2_ exists in mixed (anatase and rutile) phases at 550 °C annealing conditions, TiO_2_ predominantly exists as rutile phase at 800 °C annealed G–Mn-co-doped TiO_2_. Besides, one must notice that Mn doping could induce striking variation in the quantity and nature of OV [81,82]. Electrons introduced by OV in rutile can be distributed at crystal sites and one OV in rutile can release two nominal electrons to the crystal lattice [82]. OV concentrations in the bulk and at the surface of TiO_2_ vary depending on the heat treatment and the Ti–OH; in addition, defects are expected to significantly influence the TiO_2_ optical absorption and photocatalytic performances [81]. We attribute the enhanced photocatalytic activity for 800 °C annealed G–Mn-co-doped TiO_2_ to the variations in OV concentrations and the Ti-O defects that arise because of the inclusion of Mn and G in the crystal lattice. In summary, our photocatalytic results indicate that G and Mn, whether they are introduced in the lattice or on the surface of TiO_2_, could promote the separation of photo-generated electron–hole pairs and enhance the photocatalytic performances.

## 4. Conclusions

In summary, we have carried out an extensive investigation on the microstructural, morphological, optical, and photocatalytic properties of graphene–Mn co-incorporated composites of TiO_2_ nanowires. The co-doping of graphene and Mn^2+^/Mn^3+^ partially replace Ti^4+^ ions and creates oxygen vacancies. Consequently, phase transformation and band gap tuning can be manipulated with enhanced photocatalytic properties. The photocatalytic studies confirm that the NOx degradation efficiency of rutileTiO_2_(G–Mn) NW is higher than their anatase-rich counterparts. Among the investigated materials, T(G)Mn3 (800) exhibited optimal results from the photocatalytic performance point of view. Thus, our results demonstrate the potential of developing structurally modified rutile TiO_2_ as an efficient photocatalyst. We envisage that our study will stimulate a flurry of research activities on designing schemes for robust and cheap, rutile TiO_2-_based photocatalytic materials.

## Figures and Tables

**Table 1:** Electrospun dope compositions and product designations.

Titanium Precursor	Polymer (PVP) (g)		Doping Precursors	Mn	Electrospun Products	Calcination Temperature	
TIIP (g)	Molecular Weight (40,000)	Molecular Weight (1,300,000)	Graphene (g)		Designation	550 °C	800 °C
1.5	2.4	0.40	0	0	TP	T(550)	T(800)
			0.080	0	TP(G)	T(G)(550)	T(G)(800)
			0.080	0.04	TP(G-Mn1)	T(G)Mn1(550)	T(G)Mn1 (800)
			0.080	0.08	TP(G-Mn2)	T(G)Mn1(550)	T(G)Mn2 (800)
			0.080	0.12	TP(G-Mn3)	T(G)Mn3(550)	T(G)Mn3 (880)

**Table 2:** Summary of textural parameters (lattice parameters, mass fractions) for various sample designations obtained from XRD patterns.

	Lattice Parameters						Mass Fraction (%)	
Sample Designation	d-spacing (A)	a (Å)	b (Å)	c (Å)	Unit Cell Volume (a^3^)	Crystallite Size (t) (nm)	Anatase X_A_	Rutile X_R_
T(G)Mn1(550)	3.531	3.780	3.780	9.867	140.98	18.7	79.16	20.84
T(G)Mn2(550)	3.531	3.780	3.780	9.867	140.98	24.1	72.70	27.30
T(G)Mn3(550)	-	-	-	-	-	2.58	-	-
T(550)	3.515	3.782	3.782	9.499	136.78	21.0	90.14	9.86
T(G)(550)	3.518	3.781	3.781	9.495	136.43	15.6	93.76	6.24
T(G)Mn1(800)	3.246	4.600	4.600	4.564	96.574	8.12	0.87	99.13
T(G)Mn2(800)	3.251	4.600	4.600	4.564	96.574	66.8	Trace	~100
T(G)Mn3(880)	3.251	4.600	4.600	4.564	96.574	29.9	-	100
T(800)	3.250	4.594	4.594	2.959	62.450	49.5	1.00	99.0
T(G)(800)	3.507	4.593	4.593	2.959	62.42	31.9	Trace	100

## Supplementary Materials

The following are available online at https://www.mdpi.com/2079-4991/10/3/456/s1, Figure S1: FE-SEM images of TP(G-Mn1) (b) TP(G-Mn2) and (c) TP(G-Mn3); Figure S2: TEM images of a) T(G-Mn1)(550) NW, b) T(G-Mn2)(550) NW, c) T(G-Mn3)(550) NW, d) T(G-Mn1)(800) NW, e) a) T(G-Mn2)(800) NW and f) T(G-Mn1)(800) NW; Figure S3. Core level XPS spectrum of A) T(G-Mn1)(550) NW, T(G-Mn2)(550) NW, and T(G-Mn3)(550) NW, and B) T(G-Mn1)(800) NW, T(G-Mn2)(800) NW and T(G-Mn1)(800) NW. Figure S4. Narrow scan XPS spectrum of Ti 2p, O1s and Mn 3d region for A)T(G-Mn1)(550) NW, T(G-Mn2)(550) NW, and T(G-Mn3)(550) NW. Figure S5. Narrow scan XPS spectrum of Ti2p, O1s and Mn 3d region for B) T(G-Mn1)(800) NW, T(G-Mn2)(800) NW and T(G-Mn1)(800) NW. Figure S6. Raman spectrum of T(G-Mn1)(550) NW, T(G-Mn2)(550) NW and T(G-Mn3)(550) NW. Figure S7. Raman spectrum of T(G-Mn1)(800) NW, T(G-Mn2)(800) NW and T(G-Mn3)(800) NW. Figure S8. UV-Visible diffuse reflectance spectrum of A) T(G-Mn1)(550) NW, T(G-Mn2)(550) NW and T(G-Mn3)(550) NW and B) T(G-Mn1)(800) NW, T(G-Mn2)(800) NW and T(G-Mn3)(800) NW. Figure S9. Optical band gap determination for A) T(G-Mn1) (550) NW, T(G-Mn2) (550) NW and T(G-Mn3) (550) NW and B) T(G-Mn1) (800) NW, T(G-Mn2) (800) NW and T(G-Mn3) (800) NW.
