# Supplementary material for: Manganese and Graphene Included Titanium Dioxide Composite Nanowires: Fabrication, Characterization and Enhanced Photocatalytic Activities
Source: Nanomaterials (Basel). 2020 Mar 4;10(3):456. doi: 10.3390/nano10030456 (PMC7153601; doi:10.3390/nano10030456)
Supplement: Supplementary file 1 [file nanomaterials-10-00456-s001.pdf]

Supplementary Material

Manganese and graphene included titanium dioxide composite nanowires: Fabrication, characterization and enhanced photocatalytic activities

Jun-Cheol Lee[1,#], Anantha-Iyengar Gopalan[1,#], Gopalan Saianand [2,] Kwang-Pill Lee[1] and Wha-Jung Kim[1*]

[#]These authors contributed equally to this work
[1] Daegyeong Regional Infrastructure Technology Development Center, Kyungpook National University, Daegu, South Korea; algopal99@gmail.com
[2] Faculty of Science, The University of Newcastle, Callaghan 2308, New South Wales, Australia; SaiAnand.Gopalan@newcastle.edu.au
*Correspondence: kimwj@knu.ac.kr; Tel.: +82-53-950-6335

1. Probing morphology through FE-SEM

FE-SEM images of the non-calcined G-Mn included titanate samples (TP(G-Mn1), TP(G-Mn2) and TP(G-Mn3) show nanofiber morphology with variations in diameters and length of the nanofibers (Figure S1). Typically, the statisitical average diameter of the randomly selected TP(G-Mn1), TP(G-Mn2) and TP(G-Mn3) nanofibers are 590, 890 and 140 nm, respectively. Similarly, the length of TP(G-Mn1), TP(G-Mn2) andP(G-Mn3) nanofibers are 23, 26 and 14 µm, respectively.

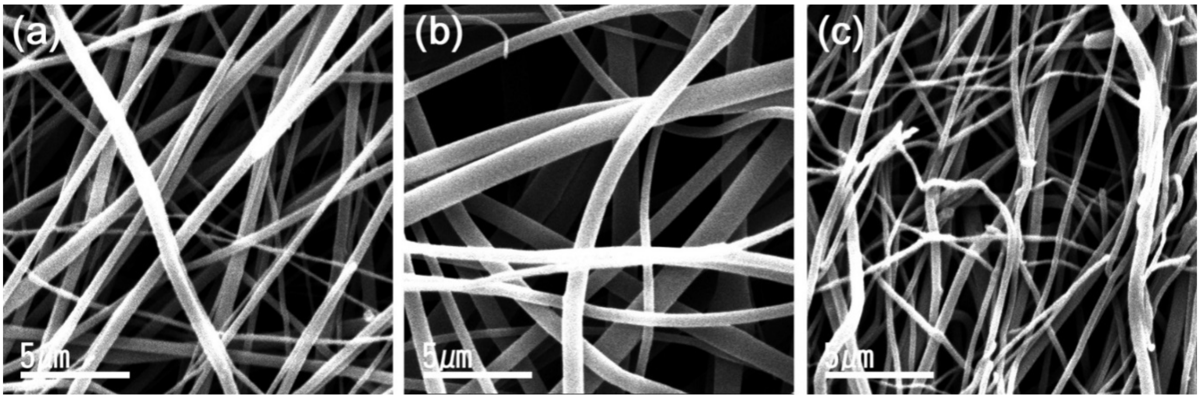

Figure S1. FE-SEM images of TP(G-Mn1) (b) TP(G-Mn2) and (c) TP(G-Mn3).

2. Transmission Electron Microscopy (TEM)

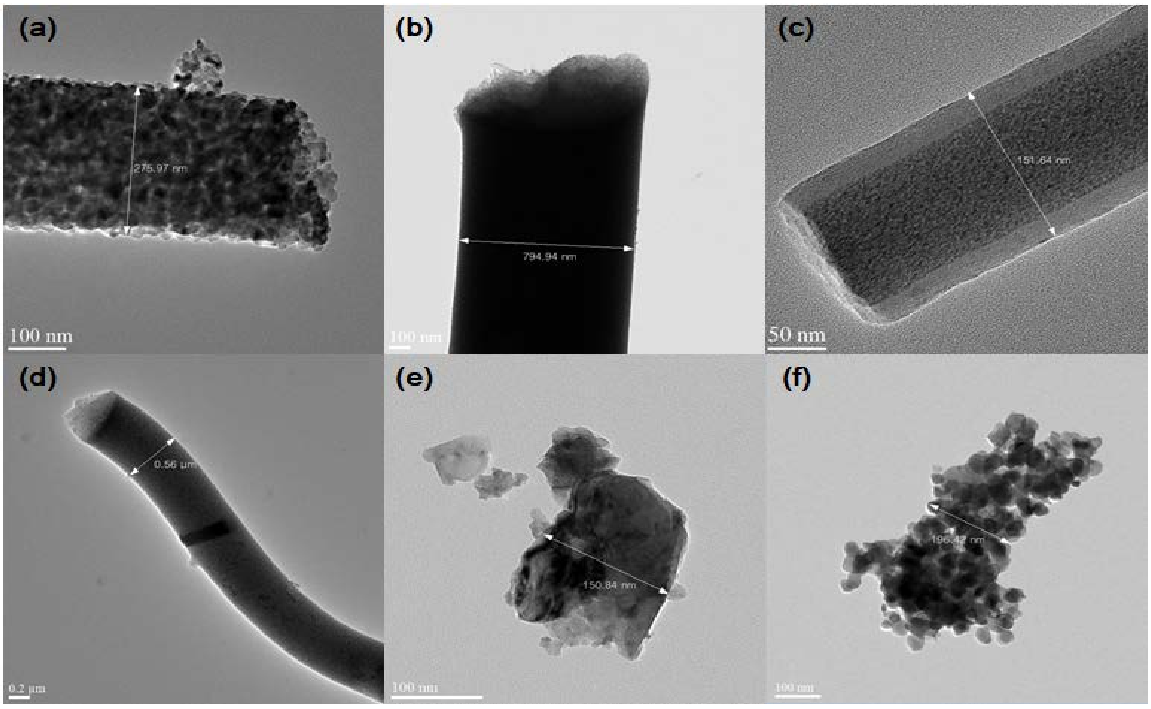

Figure S2. TEM images of a) T(G-Mn1)(550) NW, b) T(G-Mn2)(550) NW, c) T(G-Mn3)(550) NW, d) T(G-Mn1)(800) NW, e) a) T(G-Mn2)(800) NW and f) T(G-Mn1)(800) NW.

3. X-ray photoelectron spectroscopy

The chemical states of Ti, Mn, O, and C species and the interactions of graphene, Mn and TiO_2 were studied by XPS surface probe technique. The XPS survey scan analysis (Figure S3) reveals the presence of Ti, Mn, C and O through the Ti2p$^{3/2}$, Ti2p$^{1/2}$, Mn 2p$^{3/2}$ Mn2p$^{1/2}$, C1s and O1s (Figure S4) binding energy (BE) regimes. In order to obtain deeper insight onto the local chemical environments and the chemical states of atoms in the doped samples, core level and high resolution X-ray photoelectron spectra were recorded (Figure S3). As revealed in Figure S4 the Ti 2p levels (T(G)Mn1(550) had two symmetrical spin-orbit doublet peaks of Ti 2p$^{3/2}$ and Ti 2p$^{1/2}$ located at 458.18 and 463.98eV which correspond to the Ti^{4+} oxidation state of anatase TiO_2.[1] The separation of Ti 2p$^{3/2}$ and Ti 2p$^{1/2}$ binding energy is 5.80 eV. Besides, a satellite peak at 471.68 eV with a BE value higher than Ti 2p$^{3/2}$ level is noticed. This satellite peak is very characteristic for TiO_2 and may corresponds to the O 2deg → Ti 3 deg transition. The Ti 2p levels of (T(G)Mn2(550) had two symmetrical spin-orbit doublet peaks of Ti 2p$^{3/2}$ and Ti 2p$^{1/2}$ located at 457.98 and 463.78,eV, with a peak separation of 5.80 eV. It must be noted that the peak position of Ti 2p$^{3/2}$ is shifted to lower BE value for T(G)Mn2(550) as compared to T(G)Mn1(550) (Figure S3). In the case of T(G)Mn3(550), the peak separation between Ti 2p$^{3/2}$ and Ti 2p$^{1/2}$ level is increased to 6.0 eV, with BE values as 458.18 eV and 464.18 eV, respectively, for Ti 2p$^{3/2}$ and Ti 2p$^{1/2}$ levels. The shift to lower BE compared to TiO_2 can be ascribed to the increment in the electron density of Ti atom in TiO_2, indicating the probable formation of Ti–O–C bonds on the surface of TiO_2.[2] Also, the occurrences of chemical shift or difference in the peak separation between Ti 2p$^{3/2}$ and Ti 2p$^{1/2}$ levels, suggest that Mn doping can also influence the local chemistry of the host Ti atoms in the TiO_2 phase. The peak positions of Ti 2p$^{3/2}$ and Ti 2p$^{1/2}$ that correspond to samples, T(G)Mn2(800) and T(G)Mn2(800) are at 457.78 eV and 463.48 eV, respectively, that form the characteristics of pure rutile phase. The peak separation between Ti 2p$^{3/2}$ and Ti 2p$^{1/2}$ is 5.70 eV and the peak positions are slightly toward lower BE values. The satellite peak appears at 471.28 eV which is 13.5 eV away form the BE value of Ti 2p$^{3/2}$ level. T(G)Mn2(800) sample has Ti 2p$^{3/2}$ and Ti 2p$^{1/2}$ level peaks at 559.08 and 464.78 eV,

respectively, probably due to the existence of smaller portion of anatase phase. The absence of any shoulder peak for the Ti $2p^{3/2}$ level in all the samples informs the fewer probability for the co-existence of Ti^{3+} state.

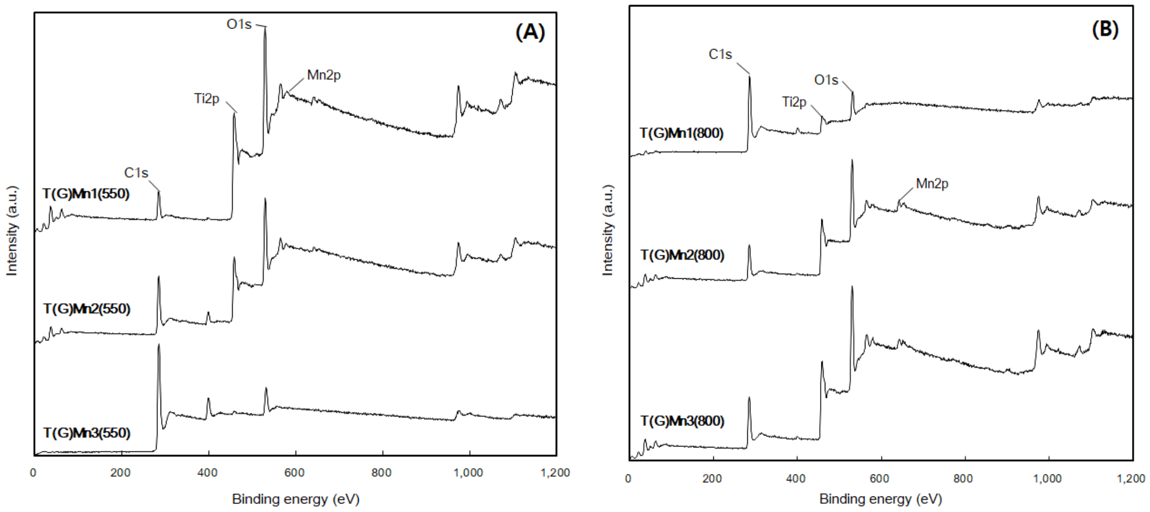

Figure S3. Core level XPS spectrum of A) T(G-Mn1)(550) NW, T(G-Mn2)(550) NW, and T(G-Mn3)(550) NW, and B) T(G-Mn1)(800) NW, T(G-Mn2)(800) NW and T(G-Mn1)(800) NW.

To investigate the chemical state of Mn species in the samplesin the form of dopants to substitute the lattice position of Ti or in the form of isolated Mn compounds, we utilized the slow scan of core level Mn 2p level XPS spectra. The T(G)Mn1(550) exhibited a 12.1 eVspaced Mn $2p^{3/2}$ and $2p^{1/2}$ peaks located at 640.88 eV and 652.78 eV, indicating the presence of Mn^{2+} or Mn^{3+} species. It must be noted that Mn $2p^{3/2}$ peak for the T(G)Mn1(550) is present at 640.88 eV, a value close to Mn^{2+} in MnO (Mn $2p^{3/2}$, 640.7 eV) in accordance with the literature, indicating a divalent state of the Mn ions in T(G)Mn1(550). Besides, it is known from literature that Mn $2p^{3/2}$ peaks for metallic Mn, Mn_3O_4, Mn_2O_3 and MnO_2 are located at 639, 641.2, 641.6 and 642.2 eV, respectively. The absence of these peaks in T(G)Mn1(550) sample excludes the possible co-existance of Mn oxides or metallic manganese clusters. The incorporation of Mn dopants in Ti lattice is expected to modify the surface energy of certain faces through preferential incorporation of Mn in some specific surfaces. XPS spectra are consistent with the presence of Mn^{2+} substituting Ti^{4+} in the TiO_2 lattice, creating oxygen vacancies in order to maintain charge neutrality. The smaller ionic radii of Mn cations in Mn^{2+} and Mn^{3+} states allows the interstitial incorporation of the dopant into the titania network which in turn produce a strain in the titania lattice which is evident through the displacement of the (101) reflection of the XRD patterns (Figure 1).[3] The BE of $Mn2p^{3/2}$ is found to match with the Mn^{2+} binding energy in MnO.[4] This indicates a divalent sate of the Mn and confirms the successful substitution of the Mn^{2+}atthe tetrahedral sites surrounded by the O_2ions. XRD data also suggests that the incorporation of Mn in the lattice preserve the anatase phase.

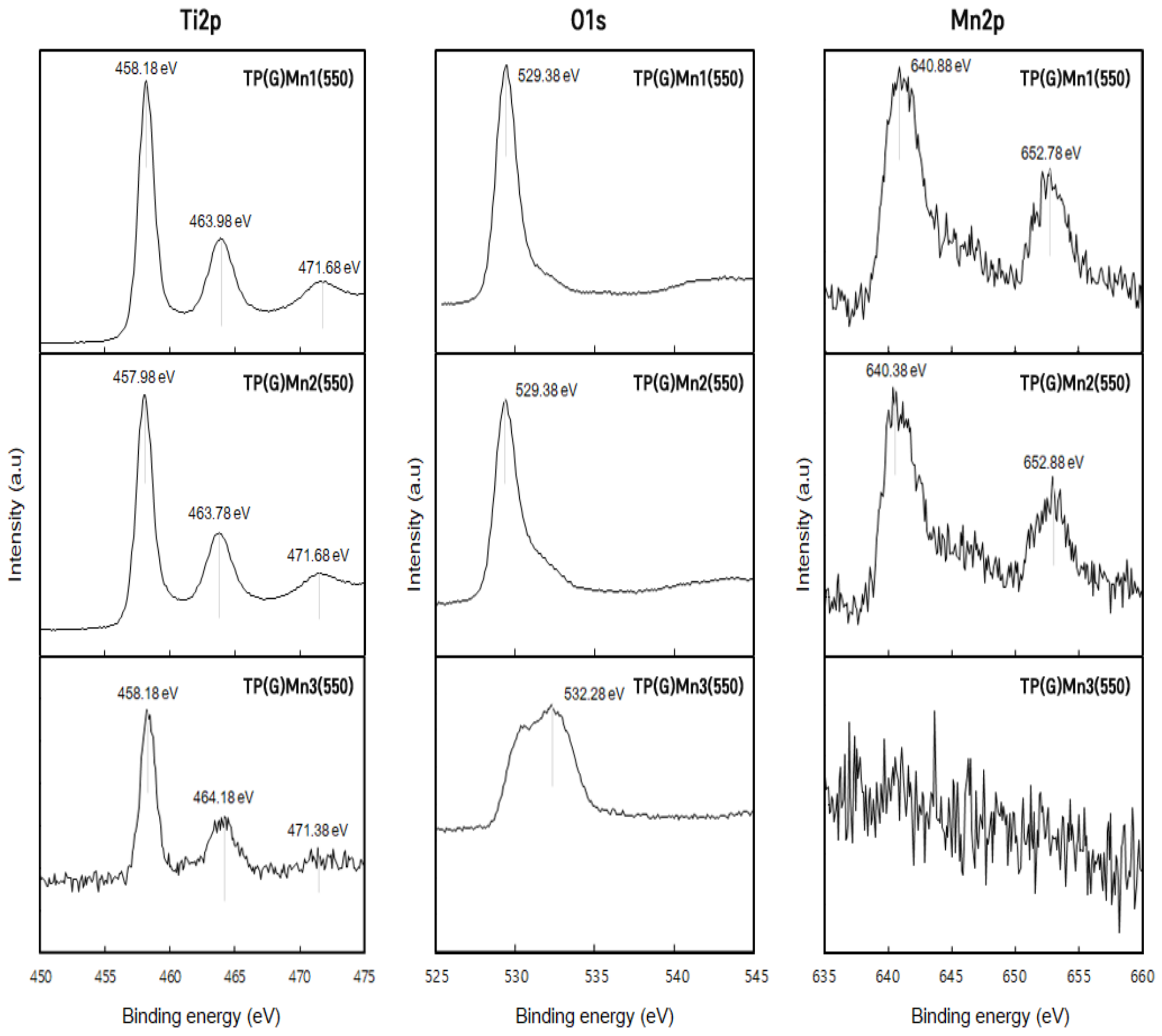

Figure S4. Narrow scan XPS spectrum of Ti 2p, O1s and Mn 3d region for A)T(G-Mn1)(550) NW, T(G-Mn2)(550) NW, and T(G-Mn3)(550) NW.

The core level XPS spectrum of sample T(G)Mn2(550) (Figure S4) shows the presence of peaks located at 640.38 and 652.88 eV corresponding to Mn $2p^{3/2}$ and $2p^{1/2}$ levels, respectively. Importantly, the BE of Mn $2p^{3/2}$in sample T(G)Mn2(550) is 0.5 eV is lower as compared to T(G)Mn1(550). And, the BE of Mn $2p^{3/2}$in sample T(G)Mn2(550) is 0.1 eV lower as compared to T(G)Mn1(550), which makes the peak separation between Mn $2p^{3/2}$ and $2p^{1/2}$ levels as 12.5 eV. The shift in the peak positions for the Mn $2p^{3/2}$ and $2p^{1/2}$ levels and difference in peak separation suggests that the incorporation of Mn dopant amount influence the crystal structure. Interestingly, there is no traceable XPS peak for Mn species at the surface of T(G)Mn3(550). At this juncture, one must remember that T(G)Mn3(550) is amorphous as inferred from the XRD data (Figure 1). It has been earlier reported that the thermal decomposition of manganese acetate at temperatures more than 600°C leads to the formation of Mn_2O_3possibly due to the oxidation/ reduction of Mn ions. Therefore, it is expected that samples of this work that were annealed at 800°C can contain most of the manganese in the gas phase is in the Mn^{3+} charge state. XPS spectrum of T(G)Mn1(800) shows BE peaks at 642.28 eV and 652.58 eV that correspond to Mn $2p^{3/2}$ and $2p^{1/2}$ levels with a spin-orbit peak separation of 10.30 eV. We attribute theincorporation of Mn^{3+} ions into the rutile TiO_2 lattice structure. The extent of Mn^{3+} inclusion into the TiO_2 lattice depends on the Mn amount selected for doping as evident from the shifts in BE of Mn $2p^{3/2}$ and $2p^{1/2}$ levels as well as the differences in the spin-orbit peak separation between samples, T(G)Mn1(800), T(G)Mn2(800) and T(G)Mn3(800).

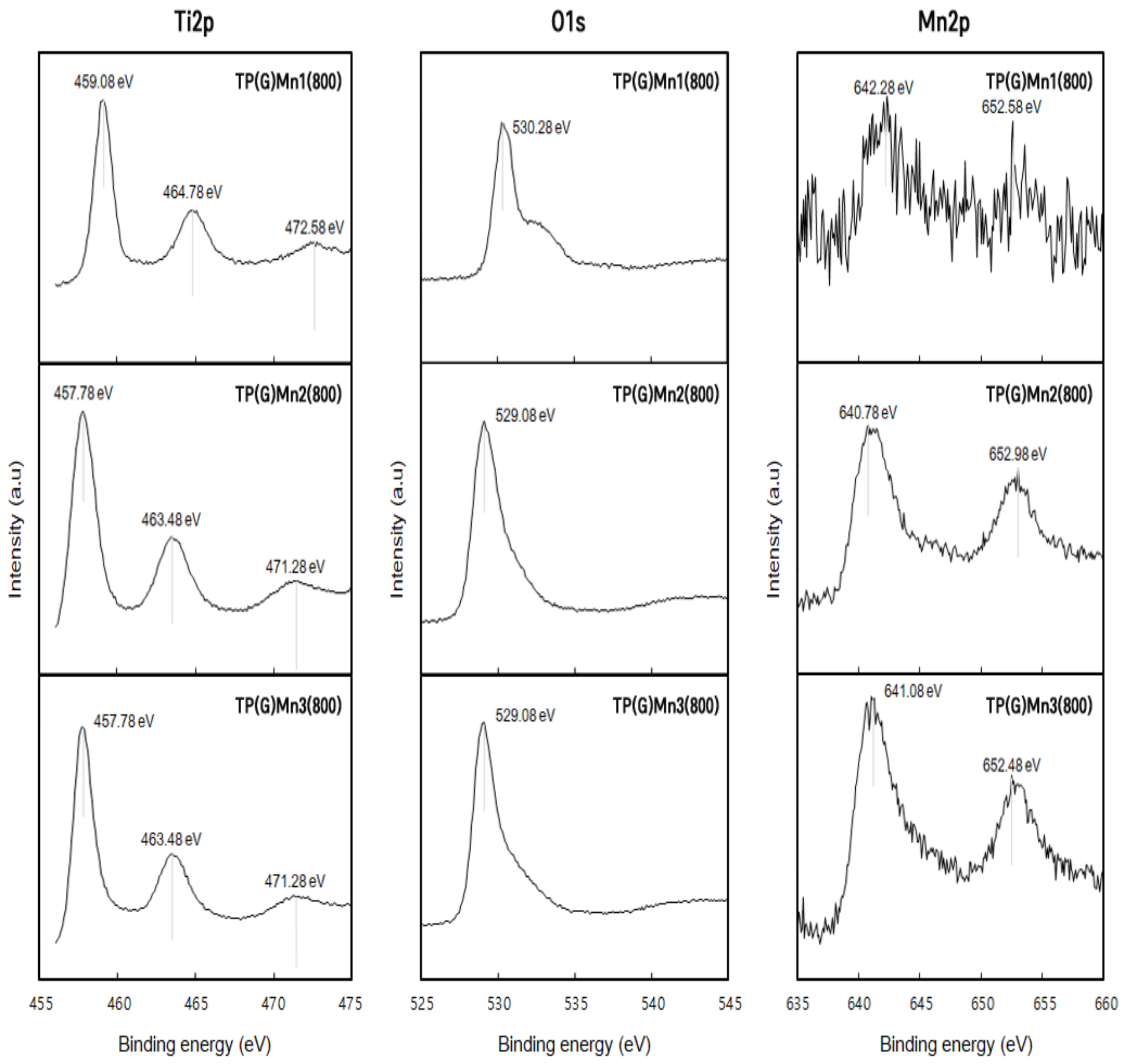

Figure S5. Narrow scan XPS spectrum of Ti2p, O1s and Mn 3d region for B) T(G-Mn1)(800) NW, T(G-Mn2)(800) NW and T(G-Mn1)(800) NW.

The O1s spectra of carbon and Mn co-doped and annealed (550°C and 800°C) samples (Figure S4 and S5) show a major peak around 529 to 532 eV, which is assigned to the oxygen bound to tetravalent Ti ions. The wide and asymmetric nature of the main peak of O 1s spectra of the samples inform that there can be more than one chemical environment for oxygen. The BE difference of ~ 71 eV between the observed peaks positions of Ti $2p^{1/2}$ and O 1s corroborates with literature.[5] O1s spectra also show a shoulder around 535-540 eV implying that the surface is partially covered with hydroxide OH groups and/or existence of some carbon/Mn included chemical environment.[6] The variations in the O1s main and shoulder peak positions suggest the possible difference in the oxygen chemical environment due to the incorporation of carbon/Mn in different proportions. Particularly, the O1s spectrum of T(G)Mn3(550) (Figure S4) exhibits a doublet type major peak around 530-532 eV, suggesting a different type of chemical environment for oxygen as compared to the other samples and this observation should be correlated to the amorphous nature of T(G)Mn3(550) as inferred through XRD analysis (Figure 1). The samples excepting the pristine TiO_2 NW show the sp^2-hybridized carbon peak appeared near ~ 284 eV with a well-known asymmetric shape. The peak position and shape of C 1s spectra of the samples are found to vary indicating the possible variations in carbon chemical environments.

4. Raman spectroscopy

The effects of carbon-Mn co-doping on the TiO_2 lattices are evaluated using Raman spectroscopy by recording the room temperature Raman spectra of various carbon-Mn co-doped various samples. Raman spectra of T(G)Mn1(550) and T(G)Mn2(550) samples (Figure S6) exhibited three B1g Raman bands with a strong peak around 148.9 cm^{-1} that corresponds to anatase phase and a weak band around 430 cm^{-1}(Eg) for the rutile structure in the spectral range between 100 and 800 cm^{-1}. The other two Eg modes are less intense Raman modes. The results are in good agreement with the co-existence as anatase and rutile crystalline phases as inferred from XRD patterns. (Figure 1, Table 1) The intensity of the strong phonon peak at 148 cm^{-1} significantly increased with a small shift for T(G)Mn2(550) as compared to T(G)Mn1(550) sample, indicating the enhancement of the sample crystallinity. On the contrary, Raman spectrum of T(G)Mn3(550) shows a prominent peak at 144, 398, 517, and 639 cm^{-1} which are characteristics of the Eg, B1g, A1g or B1g, and Eg vibration modes of amorphous TiO_2, respectively.[7] The strong phonon peak at 144 cm^{-1} corresponding to an Eg mode of T(G)Mn3(550) sample shows shift in its position by about 5 cm^{-1} as compared to anatase TiO_2 of T(G)Mn1(550)and T(G)Mn2(550), suggesting the possibility of impurity accumulation in TiO_2 structure. The B1g peak and A1g + B1g peak that appeared around 397 cm^{-1} and 516 cm^{-1}, respectively, correspond to the symmetric and anti-symmetric bending vibrations of O–Ti–O bonds. The broadening, intensity variation and shifting of the Eg(1) Raman mode is ascribed to the disruption of lattice symmetry and generation of defects such as oxygen vacancies. The charge neutrality can be maintained because of the substitution of Mn at the Ti site., There would be a decrease in the number of Ti–O and Mn–O bonds due to generation of oxygen vacancies, informing the probable lattice distortion. The variation of the crystal parameters justifies such crystal structure modification. Therefore, there can be decrease in the number of Ti–O and Mn–O bonds. Thus, it is presumed that the incorporation of carbon and Mn in the TiO_2 crystal lattice could generate oxygen vacancies and contribute to the shifting, intensity variation and broadening of the Raman peaks.

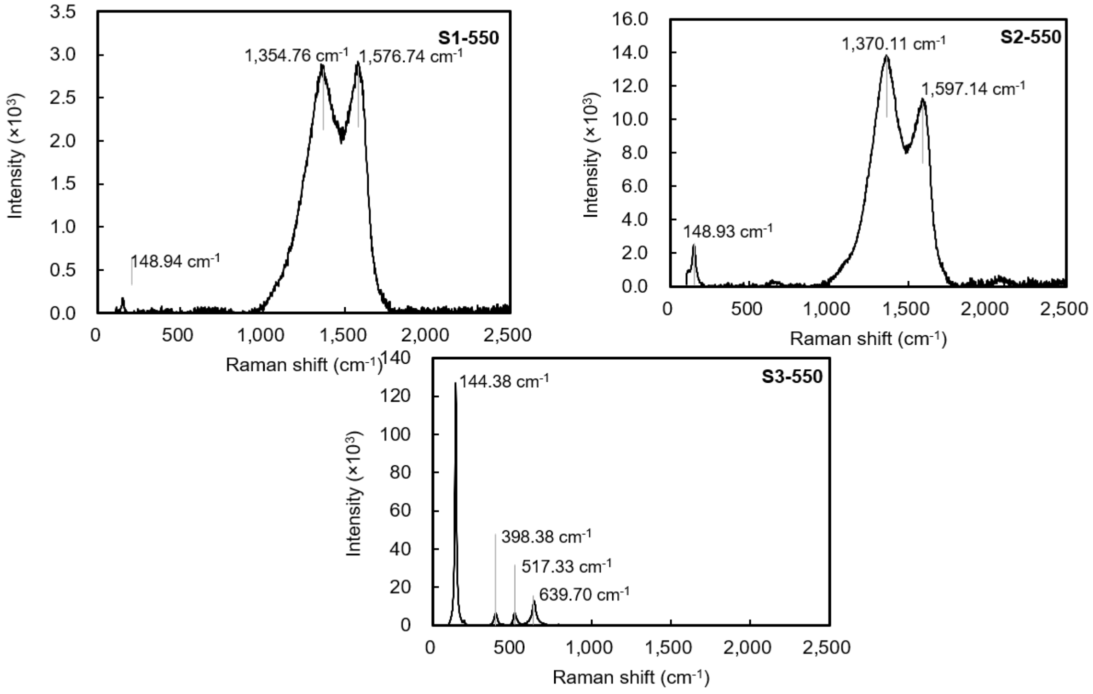

Figure S6. Raman spectrum of T(G-Mn1)(550) NW, T(G-Mn2)(550) NW and T(G-Mn3)(550) NW.

Raman spectra of G included and annealed (550°C and 800°C) samples (Figure S6 and S7) excepting T(G)Mn3(550) exhibit two characteristic Raman peaks in the frequency region around 1340–

1360 cm^{-1} and 1570–1590 cm^{-1} with variations in the peak intensities and positions, implying the influence of Mn and annealing temperature on the carbon environment in the samples. The bands at 1340–1360 cm^{-1} correspond to disordered sp^2 carbon atoms originating from the graphitic (D) environment and the bands around 1570–1590 cm^{-1}are attributed to ordered, symmetry-allowed E2g mode of sp^2 -bonded carbon atoms in extended p-conjugated graphite-like (G) arrangements. These bands are conventionally designated as D and G bands, respectively. Typically, Raman spectra of T(G)Mn1(550) shows the presence of D band at 1354 cm^{-1} and 1576 cm^{-1}, respectively. On the other hand, Raman spectrum of T(G)Mn2(550) show D and G band at 1370 and 1597 cm^{-1}, respectively, with 16 cm^{-1} and 21 cm^{-1} shifts from the D and G bands T(G)Mn1(550). The ratio of the intensities of the D and G bands (I_D/I_G) can be taken as an indication of disorder in the samples generated from defects associated with vacancies, grain boundaries, and amorphous carbons. It is clear from a comparison of I_D and I_G values and I_D/I_G ratio between T(G)Mn1(550) and T(G)Mn2(550) that upon increasing co-dopant (Mn) content in TiO_2 structure, the disorder nature in the crystal structure is enhanced. To note, the I_D/I_G values between T(G)Mn1(550) and T(G)Mn2(550) are 1.05 and 1.40 respectively. Noticeably, Raman spectrum of T(G)Mn3(550) has D and G bands with very low intensities, probably signifying the distortion of crystal structure leading to amorphous structure, which is in accordance to the XRD results (Figure 1). Raman spectra of T(G)Mn1(800), T(G)Mn2(800) and T(G)Mn3 (800) show intense D and G bands with variations in the peak positions and intensities (Figure S7). One must note that Raman spectrum of T(G)Mn1(800) exhibits the strongest D and G bands in contrast to the weaker bands noticed for T(G)Mn3(550). This observation is to be coupled with the crystalline peaks found through XRD spectrum for T(G)Mn3(800) in contrast to the amorphous XRD pattern of T(G)Mn3(800). The results suggest that annealing the sample at 800°C regenerates the rutile crystalline phase along with inclusions of C and Mn in the T(G)Mn3(800).

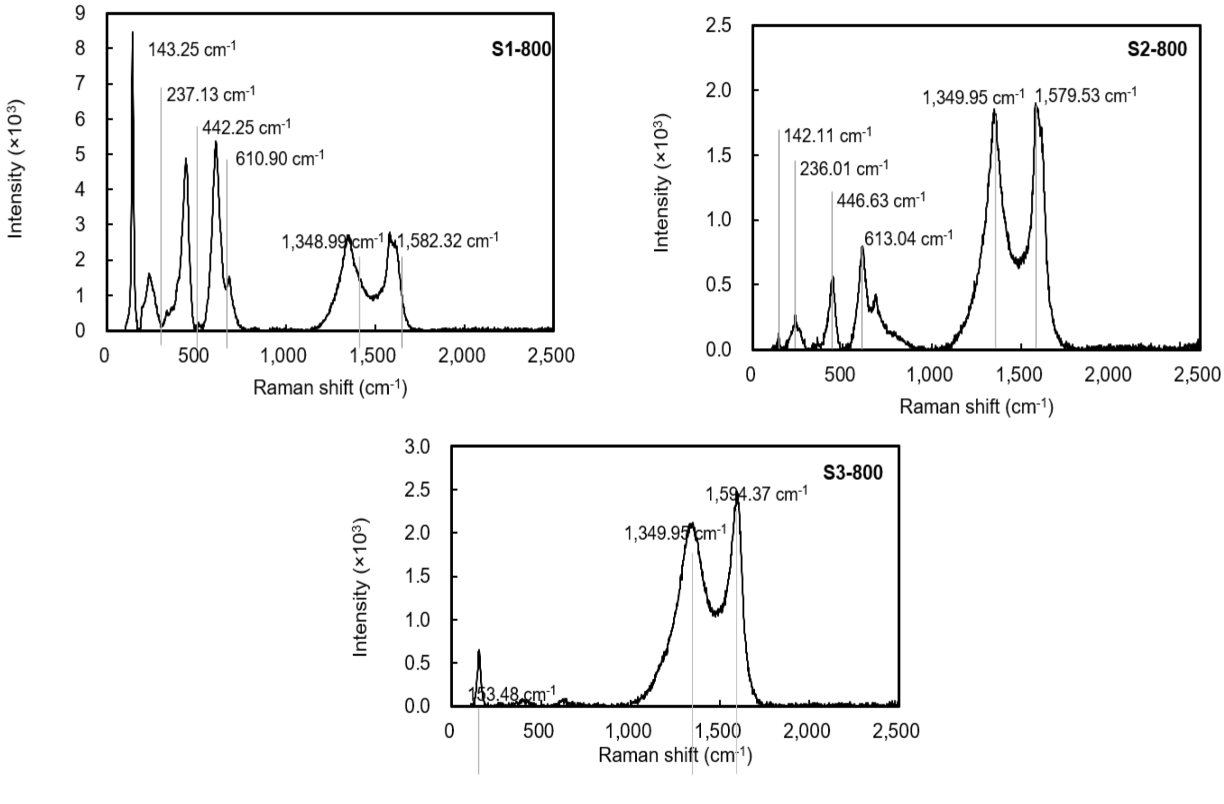

Figure S7. Raman spectrum of T(G-Mn1)(800) NW, T(G-Mn2)(800) NW and T(G-Mn3)(800) NW.

5. Diffuse reflectance spectroscopy

Figure S8 presents UV-visible diffuse reflectance spectra (DRS) of G-Mn co-doped TiO_2 NW samples in the absorption mode. One can see that the absorption edges of all G-Mn doped TiO_2NW samples are shifted to a lower energy (red shift in wavenumber) region than pristine $TiO_2$with enhanced visible region absorption. We attribute that incorporations of G and Mn into TiO_2lattice could be the reasons for the red-shifting in absorption band. Both the 550°C and 800°C calcined samples showed clear enhancement in absorption in visible region (Figure S8 (A) and (B)).We also notice that heat treatment results in the loss of band to band absorption feature and enhancement of the absorption tail in the visible region due to the formation of oxygen vacancies during annealing which creates mid-bandgap states below the conduction band (CB) leaving behind a small apparent bandgap. Calcination at 800°C, resulted in rutile formation as inferred from the XRD results and hence expected to have a lower optical band gap than anatase TiO_2. Besides, inclusion of Mn and G causes generation of interstate energy levels between CB and VB of TiO_2 and could result in lowering of band gap.

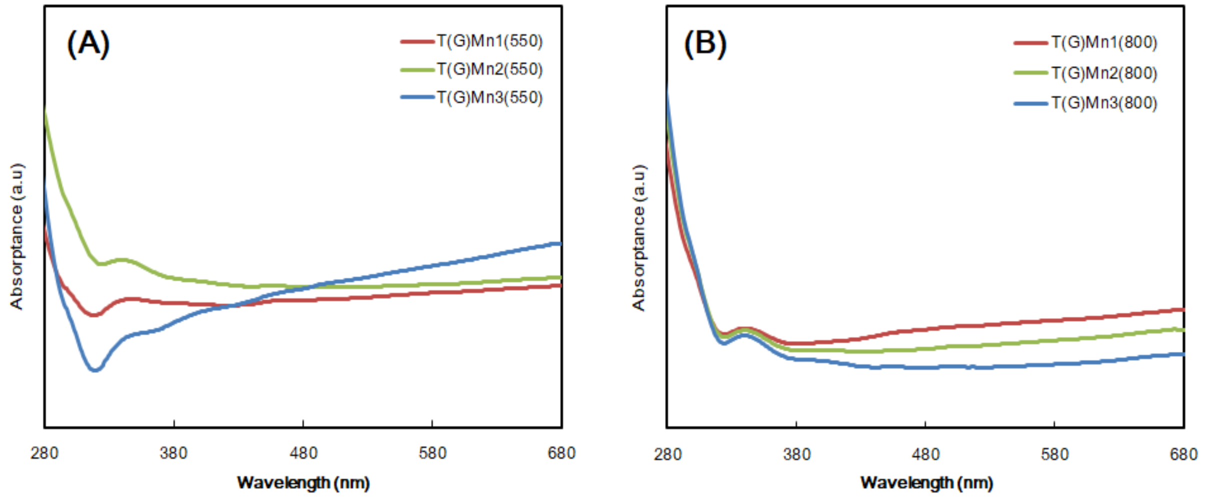

Figure S8. UV-Visible diffuse reflectance spectrum of A) T(G-Mn1)(550) NW, T(G-Mn2)(550) NW and T(G-Mn3)(550) NW and B) T(G-Mn1)(800) NW, T(G-Mn2)(800) NW and T(G-Mn3)(800) NW.

Kubelka–Munk (K-M)/Tauc plot was used to determine the optical band gap (Eg) from the DRS measurements for 550°C and 800°C annealed samples (Figure S9 (A) and (B)). The optical band gap values are obtained by extrapolating the straight line to the abscissa (X-axis) (Figure S9). The Eg values determined for the 550°C calcined samples, T(G)Mn1(550), T(G)Mn2(550) and T(G)Mn3(550) are 3.07, 3.02 and 3.67 eV, respectively (Figure S9 (A)).The Eg values of T(G)Mn1(550), T(G)Mn2(550) are lower than the anatase TiO_2 (~3.20 eV) and hence G and Mn inclusion in TiO_2further narrows the valence band (VB) and CB energy levels. Interestingly, T(G)Mn3(550) exhibits higher Eg (3.67 eV) as compared to crystalline anatase TiO_2 signifying the formation of amorphous phase as witnessed by XRD results. Amorphous anatase materials show Eg higher than 3.50 eV.[8] The Eg values of 800°C calcined samples, T(G)Mn1 (800), T(G)Mn2(800) and T(G)Mn3(800) are found to be 2.72, 2.81 and 2.90 eV, respectively. The T(G)Mn1 (800), T(G)Mn2(800) and T(G)Mn3(800) have lower Eg as compared to pure rutile (3.0 eV). Hence, G and Mn inclusion in rutile TiO_2 lattices also decrease the band gap between CB and EB. The important aspect to note is that T(G)Mn3(800) has much reduced Eg value than T(G)Mn3(550) and crystalline (Table 1, 2).

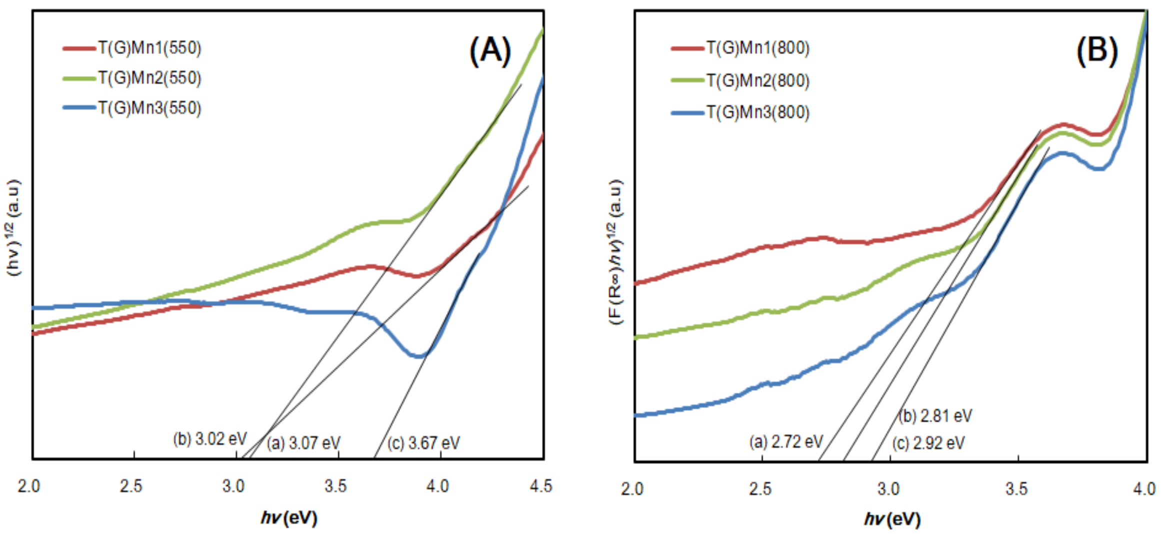

Figure S9. Optical band gap determination for A) T(G-Mn1) (550) NW, T(G-Mn2) (550) NW and T(G-Mn3) (550) NW and B) T(G-Mn1) (800) NW, T(G-Mn2) (800) NW and T(G-Mn3) (800) NW.

References

1. Xie J, Jiang D, Chen M, et al. Preparation and characterization of monodisperse Ce-doped TiO2 microspheres with visible light photocatalytic activity. *Colloids and Surfaces A: Physicochemical and Engineering Aspects.* 2010;372(1):107-114.
2. Li L, Yu L, Lin Z, Yang G. Reduced TiO2-Graphene Oxide Heterostructure As Broad Spectrum-Driven Efficient Water-Splitting Photocatalysts. *ACS Applied Materials & Interfaces.* 2016;8(13):8536-8545.
3. Wang MC, Lin HJ, Yang TS. Characteristics and optical properties of iron ion (Fe3+)-doped titanium oxide thin films prepared by a sol–gel spin coating. *Journal of Alloys and Compounds.* 2009;473(1):394-400.
4. Liu W, Tang X, Tang Z. Effect of oxygen defects on ferromagnetism of Mn doped ZnO. *Journal of Applied Physics.* 2013;114(12):123911.
5. Erdem B, Hunsicker RA, Simmons GW, Sudol ED, Dimonie VL, El-Aasser MS. XPS and FTIR Surface Characterization of TiO2 Particles Used in Polymer Encapsulation. *Langmuir.* 2001;17(9):2664-2669.
6. Iwabuchi A, Choo C-k, Tanaka K. Titania Nanoparticles Prepared with Pulsed Laser Ablation of Rutile Single Crystals in Water. *The Journal of Physical Chemistry B.* 2004;108(30):10863-10871.
7. Lambert TN, Chavez CA, Hernandez-Sanchez B, et al. Synthesis and Characterization of Titania–Graphene Nanocomposites. *The Journal of Physical Chemistry C.* 2009;113(46):19812-19823.
8. Welte A, Waldauf C, Brabec C, Wellmann PJ. Application of optical absorbance for the investigation of electronic and structural properties of sol–gel processed TiO2 films. *Thin Solid Films.* 2008;516(20):7256-7259.
